# Inflammatory bowel disease biomarkers revealed by the human gut microbiome network

**DOI:** 10.1038/s41598-023-46184-y

**Published:** 2023-11-08

**Authors:** Mirko Hu, Guido Caldarelli, Tommaso Gili

**Affiliations:** 1 Department of Medicine and Surgery, University of Parma, 43121 Parma, Italy; 2 Department of Molecular Science and Nanosystems, Ca’ Foscari University of Venice, 30123 Venice, Italy; 3 Institute of Complex Systems, National Research Council (ISC-CNR), 00185, Rome, Italy; 4 Fondazione per il Futuro delle Città, FFC, 50133 Firenze, Italy; 5 European Centre for Living Technology, (ECLT), 30123 Venice, Italy; 6 Networks Unit, IMT School for Advanced Studies Lucca, 55100 Lucca, Italy

**Keywords:** Gastrointestinal diseases, Metabolomics, Complex networks

## Abstract

Inflammatory bowel diseases (IBDs) are complex medical conditions in which the gut microbiota is attacked by the immune system of genetically predisposed subjects when exposed to yet unclear environmental factors. The complexity of this class of diseases makes them suitable to be represented and studied with network science. In this paper, the metagenomic data of control, Crohn’s disease, and ulcerative colitis subjects’ gut microbiota were investigated by representing this data as correlation networks and co-expression networks. We obtained correlation networks by calculating Pearson’s correlation between gene expression across subjects. A percolation-based procedure was used to threshold and binarize the adjacency matrices. In contrast, co-expression networks involved the construction of the bipartite subjects-genes networks and the monopartite genes-genes projection after binarization of the biadjacency matrix. Centrality measures and community detection were used on the so-built networks to mine data complexity and highlight possible biomarkers of the diseases. The main results were about the modules of *Bacteroides*, which were connected in the control subjects’ correlation network, *Faecalibacterium prausnitzii*, where co-enzyme A became central in IBD correlation networks and *Escherichia coli*, whose module has different patterns of integration within the whole network in the different diagnoses.

## Introduction

Microbes are ubiquitous. They can be found everywhere, from radioactive waste to the human gastrointestinal tract. In and on the human body, they have evolved to co-exist with their host, and it is estimated that the number of microbes hosted by the human body is of the same order of magnitude as the number of human cells^1^. In particular, the $10^{14}$ commensal microbes in the intestinal tract form the human gut microbiota, which has evolved to live in symbiosis with its host^2^. It is widely accepted that this symbiosis begins from birth, and the microbial communities stabilize with age until the formation of an adult microbiota^3^. Its genetic content (called the microbiome) characterizes everyone, also raising concerns about identity and privacy issues, specifically when the study and the manipulation of the microbiota are considered^4^. Since the 1840s, when the concept of gut microbiota first appeared, the topic has been studied for two centuries^5^, and, at the moment, it is known that the gut microbiota has a fundamental role in shaping the gut barriers^6^, training the host immune system and regulating the metabolism^7^. When the compositional and metabolic equilibrium of the commensal microbes living in the gut is disrupted, different types of diseases arise, such as metabolic disorders or central nervous system disorders^8^. Historically, traditional medicine attempted to re-establish this equilibrium through remedies intervening in the digestive system, such as fasting, diets, and the assumption of spring waters or laxatives. A recent procedure that was introduced to tackle the *Clostridioides difficile* infection is the faecal microbiota transplantation (FMT)^9^ which consists in repopulating the intestinal tract of an ill subject with the microbiota of a healthy donor.

Inflammatory bowel diseases (IBDs), which comprise Crohn’s disease (CD) and ulcerative colitis (UC), are an essential class of diseases that arise from dysbiosis and are being treated with FMT. Typical symptoms of this class of diseases are chronic diarrhoea, abdominal pain, rectal bleeding, weight loss, and fatigue^10^. Although CD and UC are both characterized by the inflammation of the intestinal tract, there are several differences between the two diagnoses that span from the environmental factors that cause them, e.g., smoking or diet, to the clinical and endoscopic findings in the two diagnoses^11^. Overall, IBDs are becoming widespread in modern society because of lifestyle changes, socioeconomic developments, and environmental causes^12^. Until now, it has been known that IBD is an exaggerated immune response to the gut microbiota of genetically predisposed subjects under the influence of the external environment. This complex interplay between genetics, the microbiota, the immune system and the environment makes it particularly hard to understand this class of diseases. Kirsner^13^ offered a complete historical review of the IBD until the 1980s, by quoting Hippocrates, who described diarrhoea as a symptom of an infectious (or non-infectious) disease to a description of the hypothetical pathogenesis of IBD, which the microbiota was not considered, though. A more recent projection predicted the evolution of the disease between 2015 and 2025 and updated the possible origins of IBD, including the action of antibiotics on the gut microbiota in Western society^14^. Xavier et al.^15^ summarized the findings of the origins of IBD, mentioning the complexity of the disease. Another historical review focuses on the genetics of the IBD^16^ identified *NOD2* as the first CD susceptible gene and then described the evolution of the IBD genetics with the coming of the modern genome-wide association study. One of the first and most comprehensive works describing the interaction of all the aforementioned factors can be found in Ref.^17^. The systems biology approach to the study of IBD was presented by Fiocchi et al.^18^, which proposed the creation of an IBD interactome, a complex system connecting all the potential agents interacting among them that derived from the combination of different omics^19^.

Our work starts from here and attempts to provide tools and methods from network science useful to build and study the IBD interactome with a systems biology approach by commencing from the metagenomic data of the gut microbiome. This approach is typical of network medicine, a novel discipline that mixes network science with systems biology to tackle the challenges offered by the progress of personalized medicine^20^, which opposes the current effective yet drastic procedures like the FMT. Network science is the discipline used to analyse complex systems. It could be suited to understand a complex disease like IBD in which a complex system like the gut microbiota plays a fundamental role. Complexity in the intestinal microbial communities arises at different scales; from the macroscopic point of view, we have the ecological interactions^21,22^ that describe the relationships among the species in the gut microbiota; among these, we have three different main types of interactions^23^; positive interactions (cooperation, commensalism, cross-feeding), negative interactions (competition, ammensalism), and asymmetric interactions (exploitation, predation, parasitism). Going towards a microscopic scale, we can find the gene networks, often represented by gene co-expression networks^24^ and metabolic networks built by connecting the substances, known as metabolites, reacting in the same metabolic processes^25^.

The application of network science for the study of the complexity of the gut microbiome is recent, and one of the first research was in the case of *C. difficile* infection^26^. The microbiome in this work was represented as a boolean network derived from binarized temporal data of the abundance of specific bacteria species in the gut. Although the study captured the dynamics of the bacterial species, e.g., negative, positive or neutral interaction, it did not take into account the genetic expression of the microbiome (metagenome), which could better explain the complex interplay between the bacterial species. Our study, by contrast, gives a static screenshot of the microbial interactions through metagenomics. A more recent study^27^ analysed the co-abundance network built with SparCC^28^; the need for this tool is due to the necessity of sparsifying the network that would have too many correlated nodes because of normalization and a *p*-value threshold too high^29^. Based on a topological property of the biological networks, the work by Vernocchi et al.^24^ portrays a weighted gene co-expression network analysis by building a network from metagenomic data and removing the weaker edges based on the assumption that the final network would be scale-free. In our work, we used thresholding methods that rely on the network topology, such as the percolation threshold or the *p*-value for the projected edges, similar to the later research. These methods should overcome the aforementioned problems. Furthermore, the emergence of specific network properties (community detection, betweenness centrality) can be used as possible IBD biomarkers. In a complex system, such as the gut microbiota, it is hard to observe changes in single biomarkers. Diseases emerge from malfunctioning in the collective behaviour of the nodes. Therefore, community detection and betweenness centralities are important properties to be analysed. The former describes how the pathways are grouped in the complex system and the latter represents the pillar pathways among all the interactions that sustain the collective functioning of the network.

## Results

In the next paragraphs, each pathway is called with a code name composed of the pathway code and the related species (e.g., “PATHWAYCODE|SPECIES”) for the sake of brevity and clarity, it is possible to consult the table mapping the correspondences between the codes and the complete pathway (Table S1 of Supplementary Material). The properties of the main pathways can be found in the same table, the BioCyc^30^ and the MetaCyc^31^ collections. The small set of reads that are mapped to proteins and are not associated with pangenomics are labelled as “unclassified”^32^.

### *Bacteroides* and *Faecalibacterium prausnitzii*

The correlation networks for each diagnosis were obtained by isolating the samples of each diagnosis and then calculating the Pearson correlation of the samples to obtain a weighted network that was made binary through a percolation threshold of each diagnosis network ($\text {th}_\text {DIAGNOSIS}$). Each correlation network was built by collecting the pathways from subjects diagnosed with Crohn’s disease (CD), ulcerative colitis (UC) or healthy non-IBD (NI). The pathways were divided into prevalent (if present in more than 75% of the samples), common (if present in 50% to 75% of the samples) and uncommon (if present in 25% to 50% of the samples). We refer to the Methods section for a more detailed description of the methods. In the prevalent pathways, the number of edges in the NI correlation network was 3356 ($\text {th}_\text {NI} = 0.453$), in CD correlation network was 2905 ($\text {th}_\text {CD} = 0.357$), and in the UC correlation network was 3160 ($\text {th}_\text {UC} = 0.364$). The results showed that the NI metagenome was more connected for prevalent pathways and the percolation threshold was higher compared to the percolation thresholds in the CD and the UC correlation networks, translating into more strongly correlated nodes in the NI correlation network. The community detection algorithm, which reveals the groups (called modules or communities) of nodes in the network based on their topological similarities, aims to maximise the modularity. The maximised modularity is an important descriptor of the network structure; the lower the modularity, the lower the total number of modules. We obtained that the lowest modularity (0.538) can be found in the NI correlation network, meaning that it was not possible to separate some of the modules and there would be interconnections between them, the CD and the UC correlation networks resulted in a modularity of 0.583 and 0.622, respectively.

**Figure 1:**
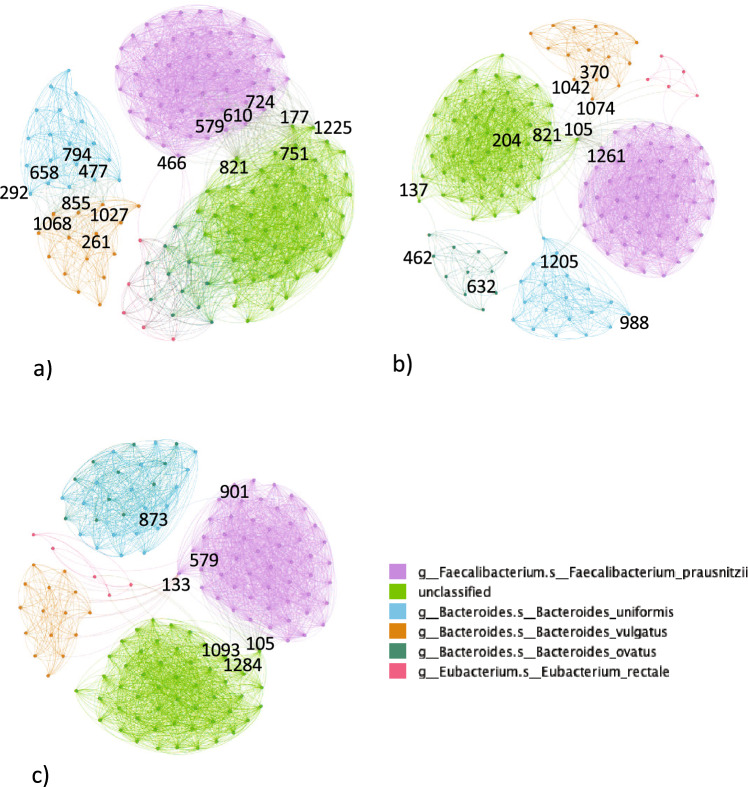
Correlation networks of prevalent metagenomic pathways in (**a**) NI subjects, (**b**) CD subjects, (**c**) UC subjects. In (**a**), nodes in the frontier between *P. vulgatus* and *B. uniformis* and in the frontier between *F. prauznitzii* and unclassified modules are highlighted. In (**b**) and (**c**), nodes with high betweenness centrality like node 105 are highlighted. The legend remarks the species of each node/pathway.

As we can see in Fig. 1, the pathways in the NI correlation network were divided into three large modules, one module was isolated, by contrast, the other two modules were communicating strictly through several nodes. The isolated module was composed of *Phocaeicola vulgatus* and *Bacteroides uniformis* pathways, this meant that in control subjects the two species co-variated and were interdependent through specific pathways (on the frontiers of the species modules, it was possible to find nodes PWY-6387|BACTEROIDES_UNIFORMIS, PWY-6151|BACTEROIDES_UNIFORMIS, PEPTIDOGLYCANSYN-PWY|BACTEIROIDES_UNIFORMIS, PWY-5667|BACTEROIDES_UNIFORMIS on the *B. uniformis* side and nodes PANTO-PWY|PHOCAEICOLA_VULGATUS, PWY-6700|PHOCAEICOLA_VULGATUS, PWY-7220|PHOCAEICOLA_VULGATUS, PWY-7222|PHOCAEICOLA_VULGATUS on the *P. vulgatus* side). The light purple module, on the other hand, contained *Faecalibacterium prausnitzii*, whereas the remaining large module contained the *Bacteroides ovatus* pathways, *Eubacterium rectale* pathways, and the unclassified species pathways. The nodes with the highest betweenness centrality (Table 1), the importance given by the number of shortest paths crossing the node, among the unclassified pathways in the two connected modules were connected through:

1. node PWY-6527|UNCLASSIFIED;
2. node GLYCOGENSYNTH-PWY|UNCLASSIFIED;
3. node PWY66-422|UNCLASSIFIED;
4. node PWY-6317|UNCLASSIFIED.

Whereas, the nodes with the highest betweenness centrality among the *F. prausnitzii* pathway module that was connected to the unclassified pathway module were:

1. node PWY-5659|FAECALIBACTERIUM_PRAUSNITZII;
2. node PWY-6277|FAECALIBACTERIUM_PRAUSNITZII;
3. node PWY-6121|FAECALIBACTERIUM_PRAUSNITZII;
4. node PWY-6122|FAECALIBACTERIUM_PRAUSNITZII.

To notice that pathway of node PWY-5659|FAECALIBACTERIUM_PRAUSNITZII correlated with only 4 *E. rectale* pathways.

**Table 1:** Top 10 pathway nodes with the highest betweenness centrality in the non-IBD (NI) correlation network with prevalent pathways.

Node	Pathway	Betweenness centrality
821	PWY-6527|UNCLASSIFIED	0.094492
466	PWY-5659|FAECALIBACTERIUM_PRAUSNITZII	0.031006
610	PWY-6122|FAECALIBACTERIUM_PRAUSNITZII	0.026071
724	PWY-6277|FAECALIBACTERIUM_PRAUSNITZII	0.026071
177	GLYCOGENSYNTH-PWY|UNCLASSIFIED	0.024356
579	PWY-6121|FAECALIBACTERIUM_PRAUSNITZII	0.017164
1225	PWY66-422|UNCLASSIFIED	0.015345
751	PWY-6317|UNCLASSIFIED	0.014598
1090	PWY-7242|FAECALIBACTERIUM_PRAUSNITZII	0.010309
499	PWY-5667|UNCLASSIFIED	0.007929

In the CD correlation network, there were fewer connections, and the network was divided into 6 modules. Each module corresponded to the species groups of pathways. The smallest module was composed of *E. rectale* pathways. The largest (light purple) module comprising *F. prausnitzii* was connected to the unclassified (green) module using node COA-PWY|UNCLASSIFIED similar to the UC correlation network. Moreover, an additional bridge connecting node was node SER-GLYSYN-PWY|FAECALIBACTERIUM_PRAUSNITZII, which was linked to nodes PWY-6527|UNCLASSIFIED and ILEUSYN-PWY|UNCLASSIFIED, to mention two high betweenness centrality nodes among the unclassified pathways. High betweenness centrality nodes DTDPRHAMSYN-PWY|UNCLASSIFIED and PWY-5659|BACTEROIDES_OVATUS connected unclassified species module with *B. ovatus* module, node PWY-6124|BACTEROIDES_OVATUS, in turn, was connected to node PWY-7219|BACTEROIDES_UNIFORMIS linking *B. ovatus* module to the *B. uniformis* module. Finally, similarly to the NI correlation network, *P. vulgatus* and *B. uniformis* were connected though (nodes PWY-3841|PHOCAEICOLA_VULGATUS, PWY-7221|PHOCAEICOLA_VULGATUS, and PWY-7228|PHOCAEICOLA_VULGATUS on the former side and nodes PWY0-845|BACTEROIDES_UNIFORMIS and 1CMET2-PWY|BACTEROIDES_UNIFORMIS on the latter side). The pathways with the highest betweenness centralities can be found in Table 2.

**Table 2:** Top 10 pathway nodes with the highest betweenness centrality in the Crohn’s disease (CD) correlation network with prevalent pathways.

Node	Pathway	Betweenness centrality
137	DTDPRHAMSYN-PWY|UNCLASSIFIED	0.419592
462	PWY-5659|BACTEROIDES_OVATUS	0.414082
632	PWY-6124|BACTEROIDES_OVATUS	0.349599
988	PWY-7219|BACTEROIDES_UNIFORMIS	0.342192
105	COA-PWY|UNCLASSIFIED	0.258219
1261	SER-GLYSYN-PWY|FAECALIBACTERIUM_PRAUSNITZII	0.133321
204	ILEUSYN-PWY|UNCLASSIFIED	0.116815
5	1CMET2-PWY|BACTEROIDES_UNIFORMIS	0.10414
1205	PWY0-845|BACTEROIDES_UNIFORMIS	0.073644
821	PWY-6527|UNCLASSIFIED	0.051347

The pathways in the UC correlation network were divided into 5 modules. The smallest module (coral red) was composed of *E. rectale* pathways scattered around the network. The dark green nodes mixed with the turquoise nodes were *B. ovatus* pathways mixed with *B. uniformis* pathways, respectively. The green module comprised of unclassified species pathways, whereas, the light purple module comprised of *F. prausnitzii* pathways. The green and the light purple modules were strictly connected similarly to the NI correlation network, the pathways connecting them were the node COA-PWY|UNCLASSIFIED, which was linked to several nodes of both modules and node PWY-6121|FAECALIBACTERIUM_PRAUSNITZII, which was linked to nodes PWY-724|UNCLASSIFIED and THRESYN-PWY|UNCLASSIFIED. Even *E. rectale* behaved as a bridge between the two large modules through a few connections. Furthermore, there was one node of *F. prausnitzii* module that was deeply correlated with all the *P. vulgatus* pathways: node DTDPRHAMSYN-PWY|FAECALIBACTERIUM_PRAUSNITZII. Finally, two nodes with high betweenness centrality were node PWY-6737|FAECALIBACTERIUM_PRAUSNITZII in the light purple module and node PWY-6703|BACTEROIDES_UNIFORMIS in the mixed dark green and turquoise module (see Table 3). Differently from the NI correlation network, *B. uniformis* did not correlate *P. vulgatus*, whereas it correlated with *B. ovatus*.

**Table 3:** Top 10 pathway nodes with the highest betweenness centrality in the Crohn’s disease (CD) correlation network with prevalent pathways.

Node	Pathway	Betweenness centrality
901	PWY-6737|FAECALIBACTERIUM_PRAUSNITZII	0.319
105	COA-PWY|UNCLASSIFIED	0.315
873	PWY-6703|BACTEROIDES_UNIFORMIS	0.311
133	DTDPRHAMSYN-PWY|FAECALIBACTERIUM_PRAUSNITZII	0.179
579	PWY-6121|FAECALIBACTERIUM_PRAUSNITZII	0.110
1093	PWY-724|UNCLASSIFIED	0.049
1284	THRESYN-PWY|UNCLASSIFIED	0.049
101	COA-PWY|FAECALIBACTERIUM_PRAUSNITZII	0.037
1008	PWY-7219|EUBACTERIUM_RECTALE	0.029
1154	PWY0-1296|UNCLASSIFIED	0.022

**Figure 2:**
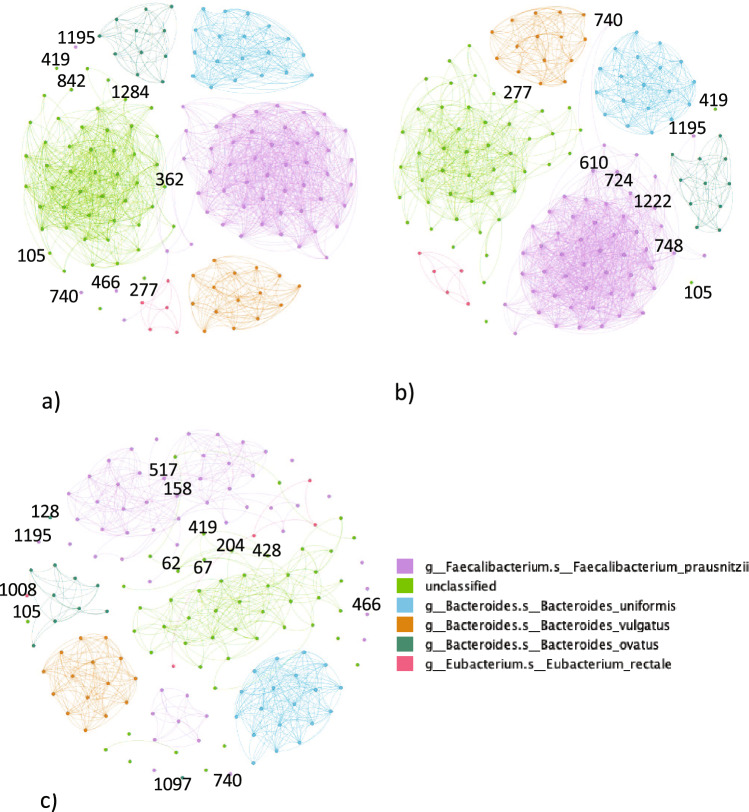
Projected network representation of prevalent metagenomic pathways in (**a**) NI subjects, (**b**) CD subjects, (**c**) UC subjects. Networks are obtained through a bipartite projection and the exclusion of an edge between two nodes is made through a comparison with a null model. The nodes highlighted describe the pathway with the highest betweenness centrality. The legend remarks the species of each node/pathway.

In Fig. 2, we have projected the bipartite network, a graph with two distinct sets of nodes (namely samples and pathways), onto the nodes of the bacterial pathways and we validate the projection through a null model (with the following parameters; significance threshold $\alpha =0.05$ and family-wise error rate $fwer = none$). Again, we divided the pathways according to their presence along with the samples. We considered the case of 75% of the presence across the samples for NI subjects. We found 1715 edges for 153 nodes and the community detection resulted in 6 large communities; namely, the unclassified species community, the *F. prausnitzii* community, the *E. rectale* community, the *B. uniformis* community, the *B. ovatus* community and *P. vulgatus* community. All the communities identified were isolated, the node COA-PWY|UNCLASSIFIED was connected to the unclassified module through the nodes PWY-6609|UNCLASSIFIED, PWY-3001|UNCLASSIFIED, and THRESYN-PWY|UNCLASSIFIED. Also, nodes PWY-5100|UNCLASSIFIED and PANTO-PWY|UNCLASSIFIED were separated from the rest of the unclassified module. Considering *F. prausnitzii* module, nodes PWY0-1586|FAECALIBACTERIUM_PRAUSNITZII, PWY-6305|FAECALIBACTERIUM_PRAUSNITZII, and PWY-5659|-FAECALIBACTERIUM_PRAUSNITZII were disconnected from the rest of the module. In Table 4, the pathways with the highest centrality belonged to unclassified and *F. prausnitzii*. This can explained by the fact that the two groups of pathways are merged in a unique module.

**Table 4:** Top 10 pathway nodes with the highest betweenness centrality in the non-IBD (NI) projected network with prevalent pathways.

Node	Pathway	Betweenness centrality
362	PWY-3001|UNCLASSIFIED	0.00645
413	PWY-5097|UNCLASSIFIED	0.00534
610	PWY-6122|FAECALIBACTERIUM_PRAUSNITZII	0.00514
724	PWY-6277|FAECALIBACTERIUM_PRAUSNITZII	0.00514
544	PWY-5695|UNCLASSIFIED	0.00430
351	PWY-2942|FAECALIBACTERIUM_PRAUSNITZII	0.00421
105	COA-PWY|UNCLASSIFIED	0.00391
1284	THRESYN-PWY|UNCLASSIFIED	0.00313
499	PWY-5667|UNCLASSIFIED	0.00242
1188	PWY0-1319|UNCLASSIFIED	0.00242

In the CD case, there were 153 nodes and 1615 edges, the community detection algorithm identified 6 different modules, one for each bacterial species identified in the previous cases. All the communities were isolated without nodes connecting them. Differently from the NI case, node COA-PWY|UNCLASSIFIED does not result in connection to the rest of the unclassified module. Similarly to the NI case, node PWY-5100|UNCLASSIFIED was not isolated, whereas node PANTO-PWY|UNCLASSIFIED was well connected to the rest of the unclassified module. When *F. prausnitzii* was considered, we obtained that node PWY0-1586|FAECALIBACTERIUM_PRAUSNITZII was connected to nodes PWY-6317|FAECALIBACTERIUM_PRAUSNITZII and PWY66-422|FAECALIBACTERIUM_PRAUSNITZII, that both were well connected to the *F. prausnitzii* pathways. Also, node PWY-6305|FAECALIBACTERIUM_PRAUSNITZII differently from the NI projected network is connected to two nodes of the bacterium module, namely PWY-6122|FAECALIBACTERIUM_PRAU-SNITZII and PWY-6277|FAECALIBACTERIUM_PRAUSNITZII, which were both pathways involving 5-aminoimidazole ribonucleotide. On the other hand, node PWY-5659|FAECALIBACTERIUM_PRAUSNITZII was connected to node PWY-5695|FAECALIBACTERIUM_PRAUSNITZII and nodes, PWY-6317|FAECALIBACTERIUM_PRAUSNITZII and PWY66-422|FAECALIBACTERIUM_PRAUSNITZII, where the former was involved in the biosynthesis of urate and the latter were involved in the degradation of galactose. Similarly to the NI case, in Table 5, the pathways with the highest centrality belonged to unclassified and *F. prausnitzii*.

**Table 5:** Top 10 pathway nodes with the highest betweenness centrality in the Crohn’s disease (CD) projected network with prevalent pathways.

Node	Pathway	Betweenness centrality
1285	TRNA-CHARGING-PWY|UNCLASSIFIED	0.0108
1225	PWY66-422|UNCLASSIFIED	0.0077
42	ARO-PWY|UNCLASSIFIED	0.0066
672	PWY-6151|UNCLASSIFIED	0.0056
525	PWY-5686|UNCLASSIFIED	0.0052
748	PWY-6317|FAECALIBACTERIUM_PRAUSNITZII	0.0045
1222	PWY66-422|FAECALIBACTERIUM_PRAUSNITZII	0.0045
865	PWY-6700|UNCLASSIFIED	0.0045
298	PEPTIDOGLYCANSYN-PWY|FAECALIBACTERIUM_PRAUSNITZII	0.0031
610	PWY-6122|FAECALIBACTERIUM_PRAUSNITZII	0.0031

In the UC case, we could find 153 nodes and 669 edges. Differently from the previous cases, the community detection algorithm could not isolate modules corresponding to the 6 species of the prevalent pathway group. The *F. prausnitzii* module was split into two modules held together by the nodes GLCMANNANAUT-PWY|FAECALIBACTERIUM_PRAUSNITZII and PWY-5686|FAECALIBACTERIUM_PRAUSNITZII. Similarly to the CD projected network, node COA-PWY|UNCLASSIFIED was isolated from the rest of the unclassified module, whereas node PWY-5100|UNCLASSIFIED was connected to the rest of the module by means of nodes BRANCHED-CHAIN-AA-SYN-PWY|UNCLASSIFIED, ILEUSYN-PWY|UNCLASSIFIED, PWY-5103|UNCLASSIFIED, and CALVIN-PWY|UNCLASSIFIED. Differently from the NI projected network and similarly to the CD projected network, in the UC projected network, the node PANTO-PWY|UNCLASSIFIED was well connected to the rest of the unclassified module. When the *F. prausnitzii* module was considered, nodes PWY0-1586|FAECALIBACTERIUM_PRAU-SNITZII, PWY-6305|FAECALIBACTERIUM_PRAUSNITZII, and PWY-5659|FAECALIBACTERIUM_PRAUSNITZII were isolated from the rest of the module in the same fashion as the NI case. Furthermore, the UC projected network by displaying fewer connections had more isolated pathways compared to the NI and the CD case. For instance, nodes DTDPRHAMSYN-PWY|BACTEROIDES_OVATUS and PWY-7282|BACTEROIDES_OVATUS detached from the *B. ovatus* module, node PWY-7219|EUBACTERIUM_RECTALE detached from the *E. rectale* module, and several more fragments of the module. Similar to the previous two cases, the highest betweenness centrality nodes belonged to *F. prausnirzii* and unclassified groups Table 6.

**Table 6:** Top 10 pathway nodes with the highest betweenness centrality in the ulcerative colitis (UC) projected network with prevalent pathways.

Node	Pathway	Betweenness centrality
67	CALVIN-PWY|UNCLASSIFIED	0.0192
158	GLCMANNANAUT-PWY|FAECALIBACTERIUM_PRAUSNITZII	0.0132
517	PWY-5686|FAECALIBACTERIUM_PRAUSNITZII	0.0132
359	PWY-2942|UNCLASSIFIED	0.0113
525	PWY-5686|UNCLASSIFIED	0.0113
865	PWY-6700|UNCLASSIFIED	0.0113
321	PWY-1042|FAECALIBACTERIUM_PRAUSNITZII	0.0104
821	PWY-6527|UNCLASSIFIED	0.0097
244	NONOXIPENT-PWY|UNCLASSIFIED	0.0066
121	COMPLETE-ARO-PWY|UNCLASSIFIED	0.0036

### *Escherichia coli*

As it is possible to observe in Fig. 4, the relatively uncommon pathways were 910 in total. There were 49203 edges in NI correlation network ($\text {th}_\text {NI} = 0.474$), 42617 edges in CD correlation network ($\text {th}_\text {CD} = 0.446$), 44523 edges in UC correlation network ($\text {th}_\text {UC} = 0.465$). The number of edges was comparable in the three networks. We could observe 9 modules in the NI correlation network (modularity 0.623), 9 modules in the CD correlation network (modularity 0.644), and 6 modules in the UC correlation network (modularity 0.695). In every network, it was possible to identify an approximately isolated ball-shaped module containing *E. coli* pathways. It was interesting to highlight the position of *B. fragilis* pathways in respect of *E. coli* pathways in the different diagnoses. In the NI correlation network, *B. fragilis* pathways were connected to the *E. coli* module through *Veillonella parvula* pathways, by contrast, in the UC correlation network, *B. fragilis* pathways were incorporated and surrounded by the same module containing *E. coli* pathways, whereas in the CD correlation network, the bacterial pathways of the two species were separated. In the NI correlation network, the module containing *E. coli* included also bacterial pathways of other species, notably *Eubacterium siraeum* and *Ruminococcus gnavus* pathways that behaved as bridge nodes between *E. coli* containing the module and the rest of the network. In the CD correlation network, this role was assumed by *Roseburia intestinalis* and *V. parvula*, whereas, in the UC correlation network, we did not observe any pathways behaving as bridge nodes. An additional important module that was also the largest was the one mainly composed of *R. torques*, *Anaerostipes hadrus*, *Lachnospiraceae bacterium 5 1 63FAA*, plus other minor species. The pathways belonging to the last two mentioned species were strictly intertwined forming the second largest ball-shaped group of nodes. The same ball-shaped group of nodes was present in the CD correlation network, by contrast, in the UC correlation network, the two species were in the same module and the nodes were not mixed in the ball but they laid separately. The highest betweenness centralities of each correlation network can be found respectively in Tables 7, 8, and 9. The NI network contains a variety of central pathways from different bacterium species, by contrast, the UC network has several central pathways belonging to *Enterocloster bolteae*.

**Table 7:** Top 10 pathway nodes with the highest betweenness centrality in the non-IBD (NI) correlation network with uncommon pathways.

Node	Pathway	Betweenness centrality
263	PANTO-PWY|RUMINOCOCCUS_GNAVUS	0.163
969	PWY-7208|EUBACTERIUM_RECTALE	0.112
1159	PWY0-1298|UNCLASSIFIED	0.043
1112	PWY-7383|UNCLASSIFIED	0.039
1007	PWY-7219|EUBACTERIUM_HALLII	0.035
836	PWY-6609|RUMINOCOCCUS_GNAVUS	0.034
819	PWY-6527|FAECALIBACTERIUM_PRAUSNITZII	0.033
756	PWY-6385|BACTEROIDES_XYLANISOLVENS	0.032
1250	RHAMCAT-PWY|ROSEBURIA_INTESTINALIS	0.031
198	ILEUSYN-PWY|RUMINOCOCCUS_OBEUM	0.028

**Table 8:** Top 10 pathway nodes with the highest betweenness centrality in the Crohn’s disease (CD) correlation network with uncommon pathways.

Node	Pathway	Betweenness centrality
156	GLCMANNANAUT-PWY|DOREA_LONGICATENA	0.180
500	PWY-5676|UNCLASSIFIED	0.072
265	PANTO-PWY|BURKHOLDERIALES_BACTERIUM_1_1_47	0.059
395	PWY-5097|BACTEROIDES_STERCORIS	0.058
247	P162-PWY|UNCLASSIFIED	0.053
913	PWY-6897|UNCLASSIFIED	0.052
1312	VALSYN-PWY|DOREA_FORMICIGENERANS	0.048
895	PWY-6737|DOREA_LONGICATENA	0.046
670	PWY-6151|ROSEBURIA_INTESTINALIS	0.043
1003	PWY-7219|DOREA_FORMICIGENERANS	0.042

**Table 9:** Top 10 pathway nodes with the highest betweenness centrality in the ulcerative colitis (UC) correlation network with uncommon pathways.

Node	Pathway	Betweenness centrality
131	DTDPRHAMSYN-PWY|ESCHERICHIA_COLI	0.175
571	PWY-6121|ENTEROCLOSTER_BOLTEAE	0.129
753	PWY-6353|UNCLASSIFIED	0.097
1096	PWY-7282|BACTEROIDES_FRAGILIS	0.097
622	PWY-6123|BACTEROIDES_FRAGILIS	0.095
178	GLYCOL-GLYOXDEG-PWY|ESCHERICHIA_COLI	0.053
599	PWY-6122|ENTEROCLOSTER_BOLTEAE	0.039
713	PWY-6277|ENTEROCLOSTER_BOLTEAE	0.039
155	GLCMANNANAUT-PWY|RUMINOCOCCUS_TORQUES	0.029
1143	PWY0-1296|ENTEROCLOSTER_BOLTEAE	0.027

In the NI network projection, we obtained a network with 910 nodes and 20078 edges. As it is possible to notice in Fig. 5, several modules emerged from the network. The most evident was the *E. coli* module which was one of the modules that composed the *E. coli* group in the uncommon pathways. This bigger module was connected to *Flavonifractor plautii* via 5 nodes on the *E. coli* side and via 5 nodes on the *F. plautii* side. On the *E. coli* side:

1. node FUCCAT-PWY|ESCHERICHIA_COLI;
2. node PWY-6737|ESCHERICHIA_COLI;
3. node PWY-6609|ESCHERICHIA_COLI;
4. node PWY-6703|ESCHERICHIA_COLI;
5. node PWY-7199|ESCHERICHIA_COLI.

On the *F. plautii* side:

1. node PWY-5188|FLAVONIFRACTOR_PLAUTII;
2. node PWY-6122|FLAVONIFRACTOR_PLAUTII;
3. node PWY-6277|FLAVONIFRACTOR_PLAUTII;
4. node PEPTIDOGLYCANSYN-PWY|FLAVONIFRACTOR_PLAUTII;
5. node PWY-6121|FLAVONIFRACTOR_PLAUTII.

Curiously there was also a *Enterocloster bolteae* pathway well-connected to the *E. coli* main module (node PWY0-1296|ENTERO-CLOSTER_BOLTEAE). The remaining part of the *E. coli* group was represented by fatty acid metabolism pathways (node FAO-PWY|ESCHERICHIA_COLI) or pathways involving mannose biosynthesis, which were connected to other species’ pathways. Apart from these nodes scattered around the network, the species group had two main ball-shaped modules thanks to the well-connected nature of the nodes inside the module. Another interesting community was the module formed by the nodes of two different species *L. bacterium 5 1 63FAA* and *A. hadrus*. The edges connecting the nodes of the two species were so dense that the two groups formed a unique module. Similarly to the NI correlation network, the NI projected network (Table 10) has diverse central pathways compared to the CD and UC projected network (Tables 11 and 12)

**Table 10:** Top 10 pathway nodes with the highest betweenness centrality in the non-IBD (NI) projected network with uncommon pathways.

Node	Pathway	Betweenness centrality
27	ARGSYNBSUB-PWY|BIFIDOBACTERIUM_LONGUM	0.153
1309	VALSYN-PWY|ENTEROCLOSTER_BOLTEAE	0.148
237	NONOXIPENT-PWY|ENTEROCLOSTER_BOLTEAE	0.142
1044	PWY-7221|BIFIDOBACTERIUM_LONGUM	0.080
878	PWY-6703|LACHNOSPIRACEAE_BACTERIUM_5_1_63FAA	0.070
1253	SALVADEHYPOX-PWY|RUMINOCOCCUS_TORQUES	0.042
891	PWY-6737|CLOSTRIDIUM_LEPTUM	0.041
660	PWY-6151|RUMINOCOCCUS_OBEUM	0.037
355	PWY-2942|LACHNOSPIRACEAE_BACTERIUM_5_1_63FAA	0.036
55	BRANCHED-CHAIN-AA-SYN-PWY|BIFIDOBACTERIUM_LONGUM	0.033

In the CD network projection, there were 910 nodes and 26505 edges. One of the immediately visible properties of the projected network was the isolated module composed of *E. coli* pathways. Compared to the NI case, there were no connections to the other species nodes. *R. torques* module was connected to *A. hadrus* module, which in turn was connected to *L. bacterium 5 1 63FAA* module. 5 nodes of *A. hadrus* community was connected to most of the nodes of the other two species exhibiting high betweenness centrality, they were:

1. node VALSYN-PWY|ANAEROSTIPES_HADRUS;
2. node PWY-5104|ANAEROSTIPES_HADRUS;
3. node PWY-5659|ANAEROSTIPES_HADRUS;
4. node PWY-6700|ANAEROSTIPES_HADRUS;
5. node PWY-6121|ANAEROSTIPES_HADRUS.

Similarly to the NI case, node GLCMANNANAUT-PWY|RUMINOCOCCUS_TORQUES was connected only to pathways of other species. Another difference with the NI projected network was the edges of *R. intestinalis* which in this case were connected to *B. instestinihominis*, whereas, in the NI case, they were connected to nodes PWY-6151|ROSEBURIA_INULINIVORANS and 1CMET2-PWY|BACTEROIDES_UNIFORMIS.

**Table 11:** Top 10 pathway nodes with the highest betweenness centrality in the Crohn’s disease (CD) projected network with uncommon pathways.

Node	Pathway	Betweenness centrality
697	PWY-621|ESCHERICHIA_COLI	0.207
81	COA-PWY-1|DOREA_LONGICATENA	0.156
680	PWY-6163|DOREA_LONGICATENA	0.098
971	PWY-7208|PARASUTTERELLA_EXCREMENTIHOMINIS	0.098
1265	SER-GLYSYN-PWY|RUMINOCOCCUS_BROMII	0.089
625	PWY-6123|BACTEROIDES_XYLANISOLVENS	0.088
584	PWY-6121|PARASUTTERELLA_EXCREMENTIHOMINIS	0.047
615	PWY-6122|PARASUTTERELLA_EXCREMENTIHOMINIS	0.047
729	PWY-6277|PARASUTTERELLA_EXCREMENTIHOMINIS	0.047
950	PWY-7111|RUMINOCOCCUS_BROMII	0.047

**Figure 3:**
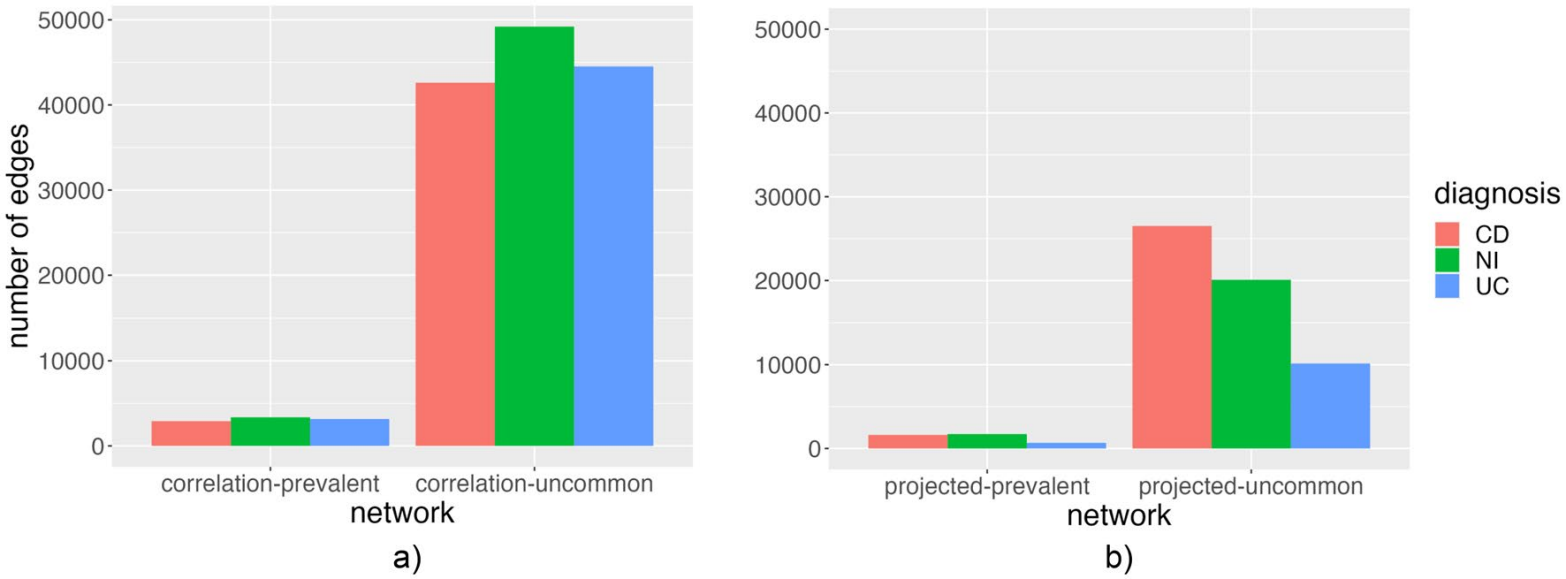
The barplots show the number of edges for each diagnosis in correlation networks **(a)** and projected networks **(b)**.

The UC network projection had 910 and only 10140 edges. The first striking property of this network was that there were much fewer edges compared to the previous two cases. It was not possible to say much about the module of *E. coli*. On the other hand, it was possible to observe that *L. bacterium 5 1 63FAA* and *A. hadrus* were separated. Furthermore, *L. bacterium 5 1 63FAA* module was connected to the *R. hominis* module through several nodes. Related to the *R. torques* group, we had one main module similar to the other cases, two isolated nodes (node PWY-7221|RUMINOCOCCUS_TORQUES as the NI case and node PWY-6121|RUMINOCOCCUS_TORQUES) and two nodes connected between them (nodes PWY-6277|RUMINOCOCCUS_TORQUES and PWY-6122|RUMINOCOCCUS_TORQUES), but separated from the rest of the pathways. As shown in Fig. 3, we found with a two-way ANOVA test a statistically significant difference in the number of edges by network type (p $< 0.00187$) for correlation networks, but not diagnosis, the interaction between these terms was not significant. On the other hand, edges had not a statistically significant difference by network type nor diagnosis for projected networks.

**Table 12:** Top 10 pathway nodes with the highest betweenness centrality in the ulcerative colitis (UC) projected network with uncommon pathways.

Node	Pathway	Betweenness centrality
314	PWY-1042|ANAEROSTIPES_HADRUS	0.0177
875	PWY-6703|BACTEROIDES_XYLANISOLVENS	0.0166
131	DTDPRHAMSYN-PWY|ESCHERICHIA_COLI	0.0109
391	PWY-5097|ANAEROSTIPES_HADRUS	0.0109
472	PWY-5667|ANAEROSTIPES_HADRUS	0.0086
1161	PWY0-1319|ANAEROSTIPES_HADRUS	0.0086
136	DTDPRHAMSYN-PWY|ROSEBURIA_INTESTINALIS	0.0079
1108	PWY-7357|ROSEBURIA_INTESTINALIS	0.0079
583	PWY-6121|ODORIBACTER_SPLACHNICUS	0.0068
320	PWY-1042|ESCHERICHIA_COLI	0.0063

**Figure 4:**
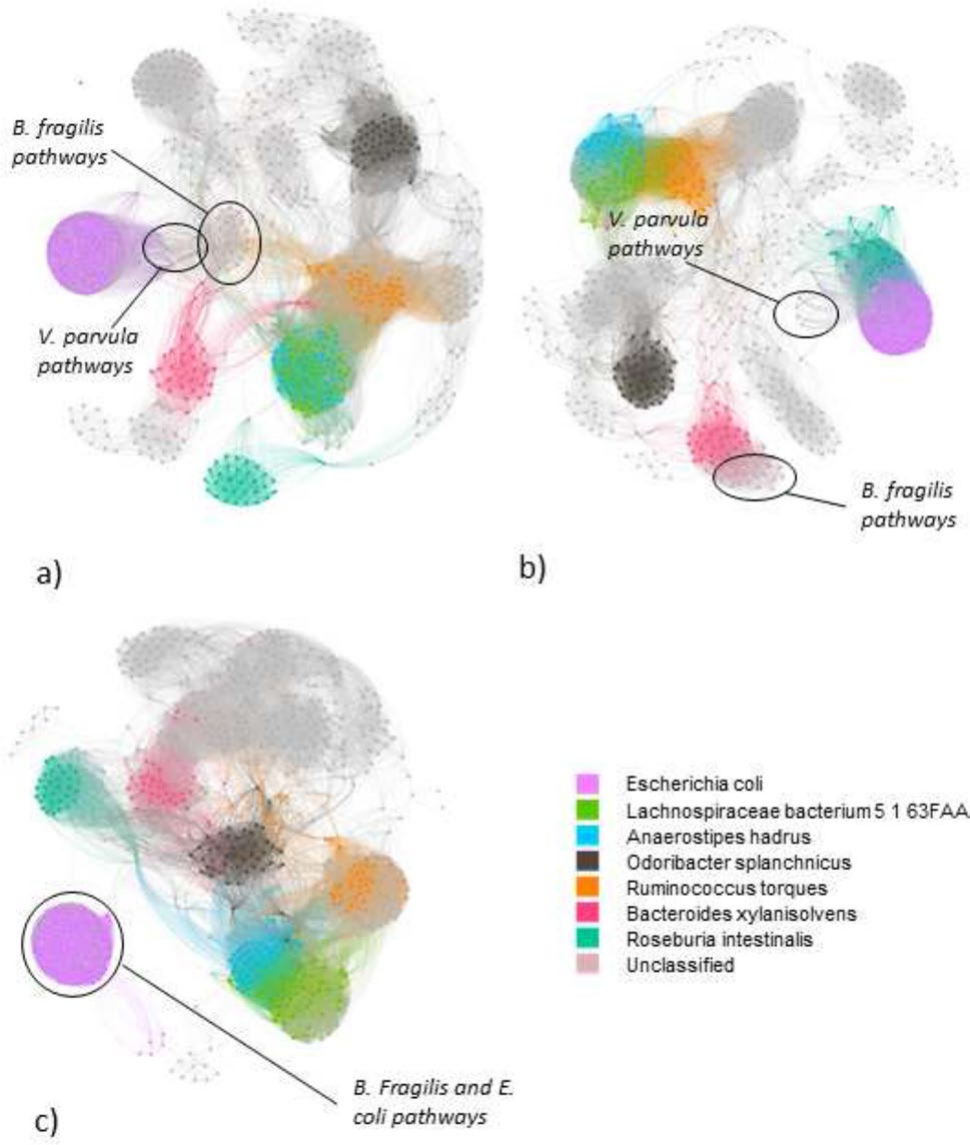
Correlation networks of uncommon metagenomic pathways in (**a**) NI subjects, (**b**) CD subjects, (**c**) UC subjects. The nodes encircled show the position of *B. fragilis* and other species like *E. coli* and *V. parvula*. The legend remarks the species of each node/pathway, where the grey nodes are classified as ’other’.

**Figure 5:**
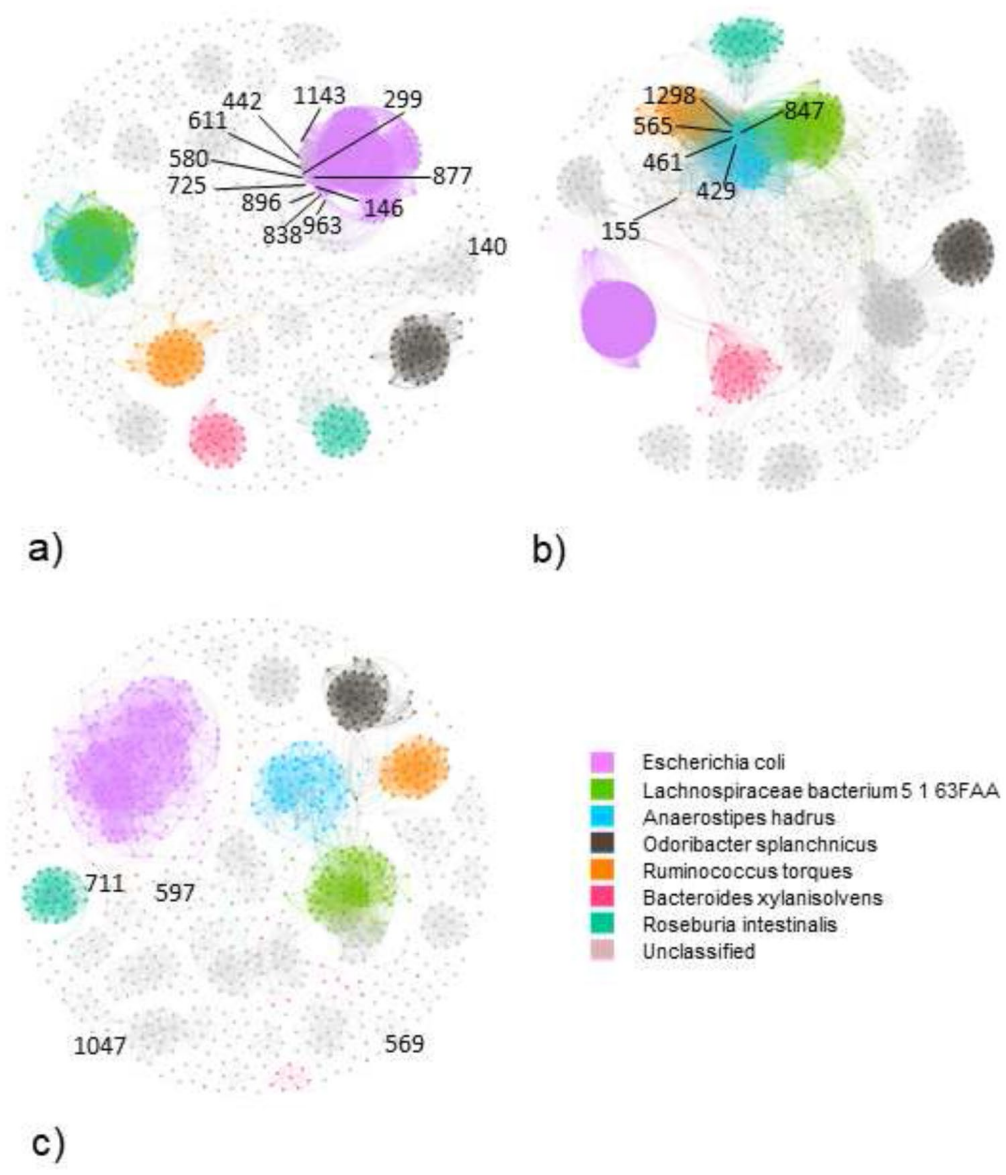
Projected network representation of uncommon metagenomic pathways in (**a**) NI subjects, (**b**) CD subjects, (**c**) UC subjects. Networks are obtained through a bipartite projection and the exclusion of an edge between two nodes is made through a comparison with a null model. The nodes highlighted correspond to nodes with high betweenness centralities or to nodes that compare to the NI projected network are isolated. The legend remarks the species of each node/pathway, where the grey nodes are classified as ’other’.

## Discussion

In the literature, the reduction of *F. prausnitzii* has been associated with IBD^33^; nevertheless, in our results, we recognized that instead of a decrease in the quantity of *F. prausnitzii* pathways expressed, there was a change in the wiring in the metagenomic network. In particular, what changed from the NI correlation network and the IBD correlation networks were the bridge pathways connecting the module of unclassified pathways and the module containing the *F. prausnitzii* pathways. For instance, in the NI correlation network, the bridge pathway was node PWY-6527|UNCLASSIFIED, whereas, in the IBD networks, the bridge pathways between the two modules were node COA-PWY|UNCLASSIFIED. The correlation between these bridge nodes and the nodes in the aforementioned modules meant that both modules relied on that specific pathway to function correctly. On the one hand, the central substance was the tetrasaccharide stachyose which is degraded into UDP-alpha-D-glucose and has been recognized as a potential probiotic against enterotoxigenic *E. coli*^34^; on the other hand, there was coenzyme A, which has a fundamental role in the metabolism and, in particular, is important in the oxidation of fatty acids. Several studies linked the alteration of fatty acid production to the IBDs; hence, this change in the centrality of the pathway related to this substance could be investigated further to explain the origins of IBDs^34^. The modules of *Bacteroides* in IBD networks corresponded to the single species, whilst in the NI network are gathered in one module. This could demonstrate that in IBDs the different *Bacteroides* species proliferates the gut independently. This fact can confirm the meta-analysis by^35^, which showed that lower levels of *Bacteroides* were associated with IBDs. Other pathways differentially wired between NI and IBD networks are those involving the bacterial metabolite 5-aminoimidazole ribonucleotide (nodes PWY-6121|FAECALIBACTERIUM_PRAUSNITZII, PWY-6122|FAECALIBACTERIUM_PRAUSNITZII, PWY-6277|FAECALIBACTERIUM_PRAUSNITZII). These nodes were behaving as bridges in the NI correlation network; by contrast, in the IBD correlation networks, they were substituted by a unique bridge node (node COA-PWY|UNCLASSIFIED).

In the range of uncommon bacterial species, we can observe the *E. coli* module; in the literature, this bacterial species has a recognized role in the development of IBD^36,37^. It was possible to observe the different interplay between *E. coli*, *V. parvula* and *B. fragilis* across the different diagnoses. The increase of *E. coli* and *B. fragilis* in IBD was observed in a previous study^38^, but our results provide additional information about the differential wiring scheme of the aforementioned species. In particular, it seemed that *V. parvula* pathways mediated the connection of *E. coli* with the other module in the correlation network. In particular, in the NI correlation network, *V. parvula* pathways were in the same module of *B. fragilis* pathways which were connected to the rest of the correlation network. In the CD correlation network, *V. parvula* pathways were included in the *E. coli* module, to remark how close the two bacterial species were, but if, on the one hand, the relationship between *E. coli* and *B. fragilis* has been already studied, the effect of *V. parvula* on *E. coli* has to be investigated yet in the literature. In the UC correlation network, *V. parvula* formed an almost completely isolated module far from the *E. coli*, this result could differentiate the connectome of the UC microbiome from the connectome of the CD microbiome. The isolation of the *E. coli* module in the UC correlation network could further represent the peculiar features of the particular form of IBD. This isolation meant that there were no correlations with the other pathways, and the metagenomic expression pattern across the samples correlated only inside the same bacterial species. In the NI correlation network, *E. siraeum* and *R. gnavus* pathways were the two main bridge pathways between *E. coli* and the rest of the network, it could be possible to hypothesize that re-establishing a connection between *E. coli* module with the aforementioned bacterial species could lead back to healthy gut microbiota. In the CD correlation network, *R. intestinalis* pathways had the role of bridge pathways, and, in fact, by using the permutation tests between the NI and CD samples, we obtained that the most differential pathways were *R. intestinalis* pathways. In the literature, this bacterial species, which has anti-inflammatory properties on the intestinal walls, was depleted in IBD subjects; nevertheless, the mechanisms underlying its protective action against IBDs are still unknown^39,40^. In the uncommon bacterial pathways of the NI projected network, the *E. coli* module was connected to the *E. bolteae*, which, in turn, was linked to *Bifidobacterium longum* module. *B. longum* is a bacterial species that can have anti-inflammatory properties in the human gut^10^. By contrast, in the CD projected network, *E. coli* was connected to the rest of the network through two *Dorea longicatena* pathways, which were nodes PWY-6163|DOREA_LONGICATENA and COA-PWY-1|DOREA_LONGICATENA and were connected to node PWY-621|ESCHERICHIA_COLI. On the other hand, in the UC projected network, two *E. coli* nodes were connected to six *C. comes* nodes, showing an interaction between the two species in the UC diagnosis.

## Methods

**Figure 6:**
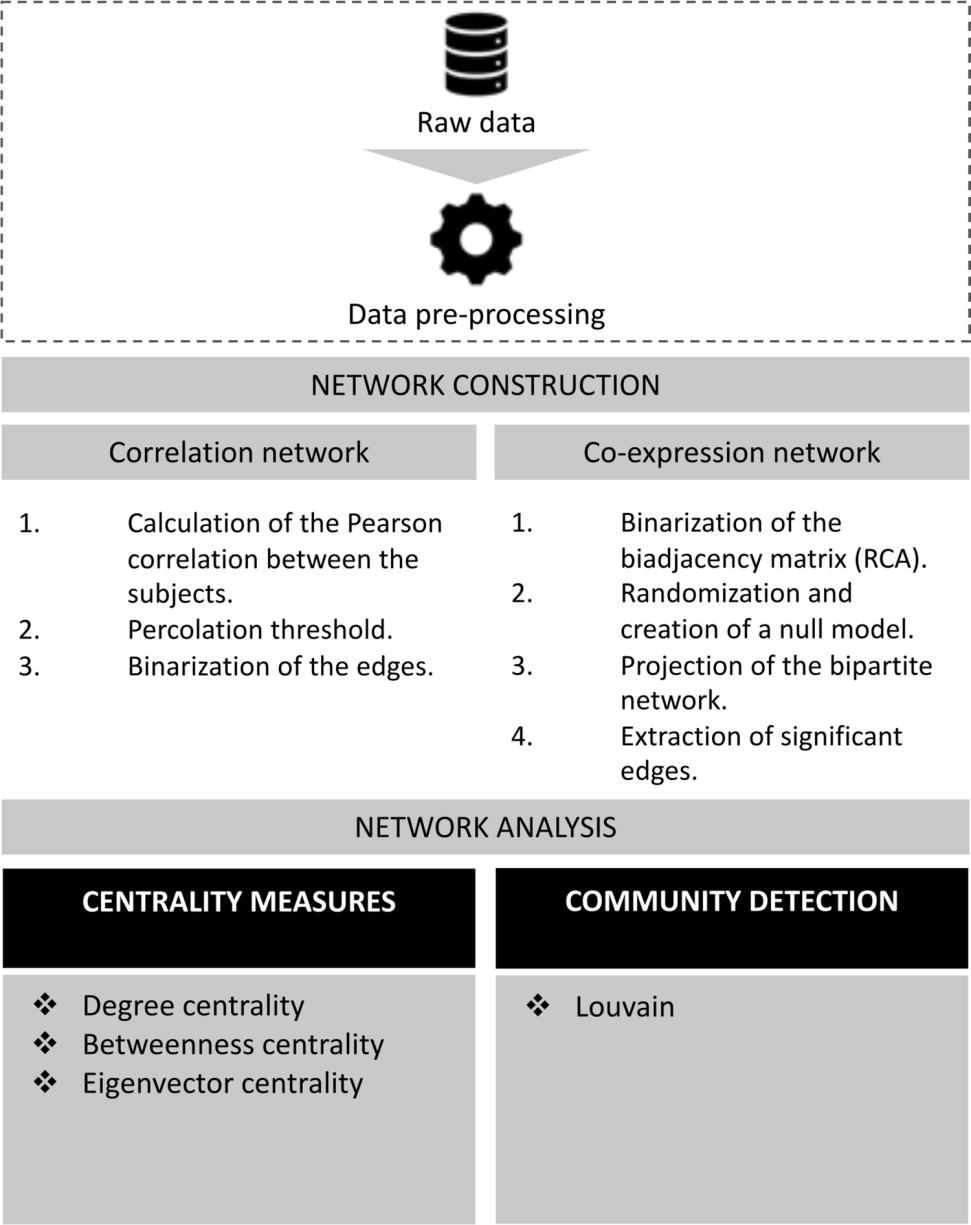
Diagram representing the methods and the network tools used to analyse the microbiome networks. The part surrounded by the dashed line was performed by the IBDMDB.

### Database

The first step of the workflow (see Fig. 6) starts from the raw data which was taken from the Inflammatory Bowel Disease Multi’omics Database (IBDMDB)^41^ is one of the first publicly available comprehensive studies of the gut ecosystem’s multiple molecular properties involved in the IBD dynamics. Some of the measurements of the microbiome offered by the study are metagenomics, metatranscriptomics and metabolomics. The data is related to 132 subjects approached in the following five medical centres: Cincinnati Children’s Hospital, Emory University Hospital, Massachusetts General Hospital, Massachusetts General Hospital for Children, and Cedars-Sinai Medical Centre. The patients recruited for the study initially arrived at the hospitals either for routine age-related colorectal cancer screening, the presence of other gastrointestinal symptoms, or suspected IBD. The latter could be a consequence of positive imaging, symptoms of chronic diarrhoea or rectal bleeding. If there were no conditions for exclusion right after enrolment, a preliminary colonoscopy was performed to determine the study strata. Based on initial analyses, the subjects that were not diagnosed with IBD (non-IBD) were labelled as ‘NI’ controls. This group of subjects included the patients who arrived for routine screening and those with more benign or non-specific symptoms^42^. The IBDMDB website contains the raw and the final results of the processed information, the complete pipeline for producing the final results is:

1. Quality and error checking for completeness, producing raw files.
2. AnADAMA pipeline, producing products.

In particular, if we consider the pipeline for producing the metagenomic data, after stool collection, the samples for the quality control process go through the KneadData^43^ and the AnADAMA pipelines. The former is a tool useful to exclude the reads, which are fragments of DNA, related to the host or related to other contaminants from the metagenomic sequencing data, and this separation step is made completely in silico. Whereas the latter, the Anadama pipeline, performs and produces documents from an automated scientific workflow, where a workflow is simply a succession of tasks, such as quantifying operational taxonomic units (OTU). The OTUs are classifications of groups of bacteria closely related to each other by sequence similarity. On the IBDMDB website, there are two versions of data Version 2.0 and Version 3.0. Version 3.0 has been uploaded with the new version of bioBakery^44^. In our work, we use the products file related to the functional profiles Version 2.0. Moreover, we exploit the HMP2 Metadata file, containing the sample IDs, the subject IDs and the properties associated with each sample. The External ID is the unique ID of the sample, Participant ID is the subject from where the sample has been taken, diagnosis is either ulcerative colitis (UC), Crohn’s disease (CD) or control group (NI), week_num points out the week number, when the sample has been taken and data_type is the type of sample (metagenomics, 16S, etc.). we extracted useful information to avoid importing the whole database, and we selected only the samples from the first week (week 0). Moreover, the samples different from metagenomic ones were excluded. Finally, we dropped the samples from the same participant in week 0 and obtained a list of samples IDs that were present in both the metagenomic database and the HMP2 Metadata. The metagenomic database contains as row indexes the gene descriptors; specifically, the descriptor is composed of the pathway, genus and species (e.g., “ARO-PWY: chorismate biosynthesis I |g__Alistipes.s__Alistipes_finegoldii”). To generate the database, the algorithm HUMAnN2^32^ has been used. The algorithm can be divided into three phases; firstly, the metagenomic sample is quickly analyzed to seek known species in the gut microbiome. The functional annotation of the identified pangenomes (i.e. the genome of a larger group of species) of the microbiome is concatenated to form a gene database of the sample. Secondly, using this database, the whole sample is aligned, meaning that statistics regarding the species and the genes are made, and unmapped reads are collected. Thirdly, the gene abundances are calculated, and they are combined with the metabolic network to determine the pathways in the microbial community.

To reduce the number of pathways present in the resulting network, we built the correlation matrices and the biadjacency matrices for the projected networks for three different groups of pathways based on quantiles; namely, one group for the pathways expressed in a percentage between 25% and 50% of the subjects (uncommon pathways), another group for those in the range between 50% and 75% (common pathways), and lastly a group for the pathways expressed in more than 75% (prevalent pathways). Originally, there were 953 nodes among the uncommon pathways. 43 nodes were disconnected from the rest of the network in the correlation networks, hence, we chose to exclude these pathways from our analysis in both the correlation and projected networks.

### Correlation Networks

Correlation networks are built from the following steps:

1. pairwise gene similarity score (correlation);
2. thresholding.

Normalization methods, correlation measures (Pearson or Spearman), significance and relevance are still debated^45^. In our work, we chose the Pearson correlation similar to Ref.^46^.

To transform a correlation matrix into a correlation network, we used a thresholding method inspired by a brain network technique that was used to cut the least important edges and keep the significant relationships among the nodes, hence, we calculated the absolute value of the correlations, making the signs irrelevant. This method consists of increasing a cut-off threshold until the network connectivity breaks apart; because of this property, this cut-off threshold is also known as the percolation threshold. This method has been considered one of the most effective methods to maximise the information quantity kept by the network^47^. In our work, we started from a cut-off threshold of $t=0$, and we used a bisection method to get to the percolation threshold. In the bisection method, we flattened the absolute values in the weighted adjacency matrix into a sorted array, chose the median value and used it as the new cut-off threshold, we calculated the connectivity of the graph built from the adjacency matrix having this cut-off threshold, finally, if we obtained a connected graph with the median value as a cut-off threshold, we used as the sorted array the upper half array, on the contrary, we used the lower half. The procedure was iterative until convergence which corresponded to an array with zero length or with the same head and same tail.

### Co-expression network from bipartite projected networks

Bipartite networks are graphs $G=(U, V, E)$ where the nodes can be divided into two disjoint sets, *U* and *V*, and every edge in *E* links an element in *U* to an element in *V*. In our work, we designated with *U* the set of nodes representing the genes and with *V* the set of nodes representing the samples. We can find in *E* all the edges connecting the gene *u* to the sample *v* if the gene was over-expressed in the corresponding sample. We evaluated the over-expression of a gene through the binarization of the data that led to the construction of a biadjacency matrix *B* of size $|U| \times |V|$ that described the bipartite network $G=(U, V, E)$ with the entries (0, 1), where $B_{ij}=1$ if the gene $v_i$ is over-expressed in the sample $u_j$, and $B_{ij}=0$ if it is not over-expressed. Biadjacency matrices are rectangular matrices where on one side there are the nodes in *U*, and on the other side the nodes in *V*.

#### Binarization

The binarization process is useful to highlight the edges in *E* that are over-expressed. By using the revealed comparative advantage (RCA)^48^, We highlighted the over-expressed genes for specific samples:

1 $$\begin{aligned} RCA_{ij}=\frac{E_{ij}/\sum _{j'\in V} E_{ij'}}{\sum _{i'\in U} E_{i'j}/\sum _{i'\in U, j'\in V} E_{i'j'}} \end{aligned}$$

where *E* is the expression of the gene *i* in the sample *j*. When $RCA_{ij}>1$, the quantity of gene *i* in sample *j* can be considered over-expressed and the entry $b_{ij}=1$ (where $b_{ij}$ is the element of the final biadjacency matrix), in the other case $RCA_{ij} \le 1$, then $b_{ij}=0$.

#### Randomization of bipartite networks

To generate a null model useful to calculate the statistically important properties of a real bipartite network, we randomized the bipartite networks by using the package BiCM^49^. In particular, the package is based on the works by Ref.^50–52^. In the aforementioned works, the Shannon entropy is defined as

2 $$\begin{aligned} \mathscr {S}=-\sum _{\textbf{M}\in \mathscr {G}}P(\textbf{M})\ln {P(\textbf{M})} \end{aligned}$$

is maximized, where $\mathscr {G}$ is an ensemble of binary, undirected, bipartite networks, and $\overrightarrow{C}(M)$ is a given set of constraints. The result is:

3 $$\begin{aligned} P\left( \textbf{M}|\overrightarrow{\theta }\right) =\frac{e^{-H\left( \textbf{M}, \overrightarrow{\theta }\right) }}{Z\left( \overrightarrow{\theta }\right) } \end{aligned}$$

Where $H\left( \textbf{M},\overrightarrow{\theta }\right) =\overrightarrow{\theta }\cdot \overrightarrow{C}(M)$ is the Hamiltonian and $Z\left( \overrightarrow{\theta }\right) =\sum _{M\in G}e^{-H\left( \textbf{M},\overrightarrow{\theta }\right) }$ is the normalization. In the case of the bipartite extension of the configuration model (BiCM), the Hamiltonian becomes:

4 $$\begin{aligned} H\left( \textbf{M},\overrightarrow{\theta }\right) =\overrightarrow{\alpha }\cdot \overrightarrow{d}(\textbf{M}) +\overrightarrow{\beta }\overrightarrow{u}(\textbf{M}) \end{aligned}$$

because we have two layers of nodes and we constrained the degree sequences $\overrightarrow{d}(\textbf{M})$ and $\overrightarrow{u}(\textbf{M})$. $\overrightarrow{d}(\textbf{M})$ is the degree sequence of the genes and $\overrightarrow{u}(\textbf{M})$ is the degree sequence of the samples.

#### Projection

One way to compress the information contained in a bipartite network is to project the bipartite network onto one of the two layers (gene/pathway layer or sample layer). We carried out the projection by connecting in the same layer the nodes that were linked by a common node in the other layer. The projection leads to a loss of information itself, so to avoid further loss of information, we weighted the edges by the number of common nodes neighbouring the nodes in the same layer^53^. The algorithm to perform the projection is:

1. select the partition on which the projection will be done
2. take two nodes of the selected partition, *n* and $n'$, and calculate their similarity
3. by evaluating the corresponding *p*-value compute the statistical significance of the calculated similarity with respect to a properly-defined null model;
4. if, and only if, the *p*-value associated with the link *n* and $n'$ is statistically significant, connect the selected nodes.

The similarity in the second step of the algorithm is evaluated by:

5 $$\begin{aligned} V_{nn'}=\sum _{c=1}^{N_c}m_{nc}m_{n'c}=\sum _{c=1}^{N_c}V_{nn'}^c, \end{aligned}$$

where $V_{nn'}^c \equiv m_{nc}m_{n'c}$ and it is clear from the definition that $V_{nn'}^c = 1$ if, and only if, both *n* and $n'$ are common neighbours of *c*. The third step of the algorithm passes through the calculation of the *p*-value of the Poisson-Binomial distribution, i.e. the probability of observing a number of V-motifs greater than, or equal to, the observed one (which will be indicated as $V_{nn'}^*$:

6 $$\begin{aligned} p-value(V_{nn'}^*)=\sum _{V_{nn'}\ge V_{nn'}^*}f_{PB}(V_{nn'}) = 1 - \sum _{V_{nn'}\le V_{nn'}^*}f_{PB}(V_{nn'}). \end{aligned}$$

Finally, in the last step of the algorithm, in order to understand which *p*-values were significant, a false-discovery rate or FDR has been adopted to take into account the fact that we were testing multiple hypotheses^54^.

### Betweenness centrality

There are many different centrality measures in network science, namely degree centrality, betweenness centrality, and eigenvector centrality; these measures describe the importance of a node in the network. In our work, we considered only the betweenness centrality, because it was the most suited to represent the importance of the pathways, i.e. the nodes. The betweenness centrality was introduced by Freeman^55^, and it considers more important the nodes that behave as bridges in the network. It can be calculated as:

7 $$\begin{aligned} C_B(i)=\sum _{s \ne t \ne i \in V} \frac{\sigma _{st}(i)}{\sigma _{st}} \end{aligned}$$

where $\sigma _{st}$ is the number of shortest paths connecting *s* and *t*, whilst $\sigma _{st}(i)$ is the number of shortest paths connecting *s* and *t* and going through *i*.

### Community detection

In the study of network science, both natural complex networks and artificial complex networks display a modular behaviour, i.e. groups of nodes are more densely connected within the members of the group than with the rest of the network. This phenomenon can also be described by a function called modularity^56^, which can be used as a parameter for one of the several ways to perform community detection in complex networks. In our work, we used the Louvain method^57^ because it is suited to large complex networks, moreover, this method is usually used when it is assumed that the gut microbiome of a healthy subject is a singular assortative network^58^. We hypothesized that the modularity changes if the network represents the gut microbiome of an IBD subject. Louvain method is based on an optimization problem that can be solved in a time $O(n \cdot log_2 n)$ where *n* is the number of nodes in the network^59^. The method is based on the aforementioned modularity optimization. Modularity is defined as in Ref.^60^,

8 $$\begin{aligned} Q=\frac{1}{2m}\sum _{i,j}\left[ A_{ij}- \frac{k_i k_j}{2m} \right] \delta (c_i,c_j). \end{aligned}$$

The algorithm is based on two phases that repeat iteratively. In the first phase, each node is repeatedly moved individually between the communities to maximize modularity. The first phase stops when no further individual move can improve the modularity. In the second phase, each community formed in the first phase is considered as a node of a weighted graph, where the weights of the edges are given by the sum of the edges connecting the nodes in the communities. The algorithm has a high efficiency partly because the gain modularity $\Delta Q$, due to moving a node *i* into a community *C*, can be steadily calculated as:

9 $$\begin{aligned} \Delta Q =\left[ \frac{\sum _{in}+k_{i,in}}{2m}-\left( \frac{\sum _{tot}+k_{i}}{2m}\right) ^2\right] -\left[ \frac{\sum _{in}}{2m} -\left( \frac{\sum _{tot}}{2m}\right) ^2 -\left( \frac{k_{i}}{2m}\right) ^2 \right] , \end{aligned}$$

where $\sum _{in}$ is the sum of the weights of the edges inside *C*, $\sum _{tot}$ is the sum of the weights of the edges going from the outside to the nodes inside *C*, $k_{i}$ is the sum of the weights of the edges going to node *i*, and $k_{i,in}$ is the sum of the weights of the edges going from *i* to the nodes in *C* and, finally, *m* is the sum of the weights of all the edges in the graph. One of the limitations of community detection based on modularity is the resolution limit^61^. This limitation to modularity may be present when $l_s \approx \sqrt{2L}$, where $l_s$ is the number of internal links in a module *S* and *L* is the total number of links in the network and it can be overcome through several methods, one of the most promising is Surprise maximization^62^. The modularity in correlation networks differs from the modularity in projected networks mainly because, in the former networks, the information being represented is based on the covariance of the pathway expressions across all the subjects, whereas, in the latter networks, the information is built on a cumulative measure that counts the number of subjects sharing common pathways. Therefore, in the first case, community detection identifies groups of correlated pathways and, in the second case, it identifies pathways that often appear in the same subject.

### Supplementary Information

Supplementary Information.

## Data Availability

The datasets used and/or analysed during the current study are available from the corresponding author on reasonable request.
