# Supplementary material for: Inflammatory bowel disease biomarkers revealed by the human gut microbiome network
Source: Sci Rep. 2023 Nov 8;13:19428. doi: 10.1038/s41598-023-46184-y (PMC10632483; doi:10.1038/s41598-023-46184-y)
Supplement: Supplementary file 1 — Supplementary Information. [file 41598_2023_46184_MOESM1_ESM.pdf]

Node	PATHWAYCODE | SPECIES	Extended metabolic pathway name	Genus	Species	Pathway description
5	1CMET2-PWY | BACTEROIDES_UNIFORMIS	N10-formyl-tetrahydrofolate biosynthesis	*Bacteroides*	*Bacteroides uniformis*	Tetrahydrofolate is also known as vitamin B9, folate coenzymes are involved in the acceptance and donation of carbon-based molecules fundamental for the nucleotide synthesis and amino acid involving pathways. 10-formyl-tetrahydrofolates are used for tetrahydrofolate, purine and formate production.
27	ARGSYNBSUB-PWY | BIFIDOBACTERIUM_LONGUM	L-arginine biosynthesis II (acetyl cycle)	*Bifidobacterium*	*Bifidobacterium longum*	The cyclic pathway for arginine biosynthesis has been demonstrated in many organisms. Arginine biosynthesis is notable for its complexity and variability at the genetic level, and by its connection with several other pathways, such as pyrimidine and polyamine biosynthesis, and certain degradative pathways.
41	ARO-PWY | ROSEBURIA_INULINIVORANS	Chorismate biosynthesis I	*Roseburia*	*Roseburia inulinivorans*	Chorismate is a relevant intermediate that leads to the production of some fundamental metabolites, among which vitamins E and K.
42	ARO-PWY | UNCLASSIFIED	Chorismate biosynthesis I	Unclassified	Unclassified	
55	BRANCHED-CHAIN-AA-SYN-PWY | BIFIDOBACTERIUM_LONGUM	Superpathway of branched amino acid biosynthesis	*Bifidobacterium*	*Bifidobacterium longum*	A number of shared enzymes connect the biosynthesis pathways for the three branched chain amino acids L-leucine, L-isoleucine and L-valine.
62	BRANCHED-CHAIN-AA-SYN-PWY | UNCLASSIFIED	Superpathway of branched amino acid biosynthesis	Unclassified	Unclassified	
67	CALVIN-PWY | UNCLASSIFIED	Calvin-Benson-Bassham cycle	Unclassified	Unclassified	The Calvin cycle is the major CO2 fixation pathway, found in many autotrophic bacteria.
101	COA-PWY | FAECALIBACTERIUM_PRAUSNITZII	Coenzyme A biosynthesis	*Faecalibacterium*	*Faelibacterium prauznitzii*	Coenzyme A (CoA) is a ubiquitous compound found in archaea, bacteria, plants, and animals. It is a common acyl carrier in prokaryotic and eukaryotic cells and is required for a multitude of reactions for both biosynthetic and degradative pathways, including the oxidation of fatty acids, carbohydrates, and amino acids.
105	COA-PWY | UNCLASSIFIED	Coenzyme A biosynthesis I	Unclassified	Unclassified	
81	COA-PWY-1 | DOREA_LONGICATENA	Coenzyme A biosynthesis II (mammalian)	*Dorea*	*Dorea longicatena*	Coenzyme A is a crucial cofactor in all living organisms. It functions as an acyl carrier and carbonyl-activating group in numerous central biochemical transformations, including the TCA cycle III (animals) and fatty acid metabolism.

121	COMPLETE-ARO-PWY | UNCLASSIFIED	Superpathway of aromatic amino acid biosynthesis	Unclassified	Unclassified	The aromatic amino acids L-phenylalanine, L-tyrosine and L-tryptophan are biosynthesized from the principal common precursor chorismate.
128	DTDPRHAMSYN-PWY | BACTEROIDES_OVATUS	dTDP-L-rhamnose biosynthesis I	*Bacteroides*	*Bacteroides ovatus*	β-L-rhamnopyranose is a deoxysugar that functions as a building block of the glycan component of the lipopolysaccharide present in ECA, the enterobacterial common antigen, and of the O-antigens of many bacterial species.
131	DTDPRHAMSYN-PWY | ESCHERICHIA_COLI	dTDP-L-rhamnose biosynthesis I	*Escherichia*	*Escherichia coli*	
133	DTDPRHAMSYN-PWY | FAECALIBAC-TERIUM_PRAUSNITZII	dTDP-L-rhamnose biosynthesis I	*Faecalibac-terium*	*Faecalibac-terium prausnitzii*	
136	DTDPRHAMSYN-PWY | ROSE-BURIA_INTESTINALIS	dTDP-L-rhamnose biosynthesis I	*Roseburia*	*Roseburia intestinalis*	
137	DTDPRHAMSYN-PWY | UNCLASSIFIED	dTDP-L-rhamnose biosynthesis I	Unclassified	Unclassified	
140	FAO-PWY | ESCHERICHIA_COLI	Fatty acid β-oxidation I	*Escherichia*	*Escherichia coli*	The catabolism of fatty acids proceeds via several routes, which depend on the length of the acids, whether the number of carbons is odd or even, and whether they are saturated or unsaturated. This pathway represents the "core" cycle of β-oxidation, a mechanism that removes two carbon atoms with each turn of the cycle.
146	FUCCAT-PWY | ESCHERICHIA_COLI	Fucose degradation	*Escherichia*	*Escherichia coli*	L-fucose can be used by some organisms as a total source of carbon and energy.
156	GLCMANNANAUT-PWY | DOREA_LONGICATENA	Superpathway of N-acetylglucosamine, N-acetylmannosamine and N-acetylneuraminate degradation	*Dorea*	*Dorea longicatena*	Amino sugars can also be utilized as carbon and nitrogen sources. N-acetylglucosamine, -mannosamine and -neuraminic acid can all be transported into the cell and metabolized.
158	GLCMANNANAUT-PWY | FAECALIBAC-TERIUM_PRAUSNITZII	Superpathway of N-acetylglucosamine, N-acetylmannosamine and N-acetylneuraminate degradation	*Faecalibac-terium*	*Faecalibac-terium prausnitzii*	
155	GLCMANNANAUT-PWY | RUMINOCOC-CUS_TORQUES	Superpathway of N-acetylglucosamine, N-acetylmannosamine and N-acetylneuraminate degradation	*Blautia*	*Ruminococcus torques*	

177	GLYCOGENSYNTH-PWY | UNCLASSIFIED	Glycogen biosynthesis I (from ADP-D-Glucose)	Unclassified	Unclassified	ADP-α-D-glucose is devoted to the synthesis of glycogen in bacteria.
178	GLYCOL-GLYOXDEG-PWY | ESCHERICHIA_COLI	Superpathway of glycol metabolism and degradation	*Escherichia*	*Escherichia coli*	*Escherichia coli* encodes the enzymes by which it can degrade ethylene glycol to glyoxylate and subsequently 3-phosphoglycerate which can enter central metabolism through glycolysis.
198	ILEUSYN-PWY | BLAUTIA_OBEUM	L-isoleucine biosynthesis I (from threonine)	*Blautia*	*Blautia obeum*	The pathway of L-isoleucine biosynthesis from L-threonine is a five-step pathway that shares its last four steps with the pathway of L-valine biosynthesis. These entwined pathways are part of the superpathway of branched chain amino acid biosynthesis, that generates not only L-isoleucine and L-valine, but also L-leucine.
204	ILEUSYN-PWY | UNCLASSIFIED	L-isoleucine biosynthesis I (from threonine)	Unclassified	Unclassified	
237	NONOXIPENT-PWY | ENTERO-CLOSTER_BOLTEAE	Pentose phosphate pathway (non-oxidative branch)	*Enterocloster*	*Enterocloster bolteae*	The pentose phosphate pathway is an important member of central metabolism, as it supplies three of the 13 precursor metabolites.
244	NONXIPENT-PWY | UNCLASSIFIED	Pentose phosphate pathway (non-oxidative branch)	Unclassified	Unclassified	
247	P162-PWY | UNCLASSIFIED	L-glutamate degradation V (via hydroxyglutarate)	Unclassified	Unclassified	Some bacteria living in anoxic environments are able to ferment amino acids to ammonia, CO2, H2, acetate and short chain fatty acids. Proteins are initially hydrolyzed by exogenous proteases to small peptides and single amino acids, which are then taken up by the bacteria and degraded internally.
265	PANTO-PWY | BURKHOLDERI-ALES_BACTERIUM_1_1_47	Phosphopantothenate biosynthesis I	*Burkholderiales*	*Burkholderiales bacterium 1 1 47*	(R)-4'-phosphopantothenate is the universal precursor for the synthesis of the 4'-phosphopantetheine moiety of coenzyme A and acyl carrier protein.
261	PANTO-PWY | PHOCAE-ICOLA_VULGATUS	Phosphopantothenate biosynthesis I	*Phocaeicola*	*Phocaeicola vulgatus*	
263	PANTO-PWY |RU-MINOCOCCUS_GNAVUS	Phosphopantothenate biosynthesis I	*Ruminococcus*	*Ruminococcus gnavus*	
277	PANTO-PWY | UNCLASSIFIED	Phosphopantothenate biosynthesis I	Unclassified	Unclassified	
292	PEPTIDOGLYCANSYN-PWY | BACTEIROIDES_UNI-FORMIS	Peptidoglycan biosynthesis I (meso-diaminopimelate containing)	*Bacteroides*	*Bacteroides uniformis*	Peptidoglycan is found on the outside of the cytoplasmic membrane of almost all eubacteria, and is unique to these organisms.

298	PEPTIDOGLYCANSYN-PWY | FAECALIBAC-TERIUM_PRAUSNITZII	Peptidoglycan biosynthesis I (meso-diaminopimelate containing)	*Faecalibac-terium*	*Faecalibac-terium prausnitzii*	
299	PEPTIDOGLYCANSYN-PWY | FLAVONIFRAC-TOR_PLAUTII	Peptidoglycan biosynthesis I (meso-diaminopimelate containing)	*Flavonifractor*	*Flavonifractor plautii*	
1143	PWY0-1296 | ENTERO-CLOSTER_BOLTEAE	Purine ribonucleosides degradation	*Enterocloster*	*Enterocloster bolteae*	Bacteria like *Escherichia coli* can use all four naturally occurring purine ribonucleosides (adenosine, guanosine, inosine and xanthosine) as total sources of carbon and energy.
1154	PWY0-1296 | UNCLASSIFIED	Purine ribonucleosides degradation	Unclassified	Unclassified	Bacteria like *Escherichia coli* can use all four naturally occurring purine ribonucleosides (adenosine, guanosine, inosine and xanthosine) as total sources of carbon and energy.
1161	PWY0-1319 | ANAEROSTIPES_HADRUS	CDP-diacylglycerol biosynthesis II	*Anaerostipes*	*Anaerostipes hadrus*	Phospholipids are important membrane components. Most of the phospholipids belong to the category of phospho-glycerides. The simplest phosphoglycerides, which are known as phosphatidates, are composed of a glycerol molecule attached to two fatty acids and one phosphate group.
1188	PWY0-1319 | UNCLASSIFIED	CDP-diacylglycerol biosynthesis II	Unclassified	Unclassified	
1195	PWY0-1586 | FAECALIBAC-TERIUM_PRAUSNITZII	Peptidoglycan maturation (meso-diaminopimelate containing)	*Faecalibac-terium*	*Faecalibac-terium prausnitzii*	The peptidoglycan (PG) is assembled from monomer sub-units of the disaccharide pentapeptide.
1205	PWY0-845 | BAC-TEROIDES_UNIFORMIS	Superpathway of pyridoxal 5'-phosphate biosynthesis and salvage	*Bacteroides*	*Bacteroides uniformis*	This superpathway shows the various ways that bacteria like *Escherichia coli* can obtain pyridoxal 5'-phosphate, a coenzyme for many enzymes that participate in amino acid and glycogen metabolism.
314	PWY-1042 | ANAEROSTIPES_HADRUS	Glycolysis IV (plant cytosol)	*Anaerostipes*	*Anaerostipes hadrus*	Glycolysis, which was first studied as a pathway for the utilization of glucose, is one of the major pathways of cen-tral metabolism, the other two being the pentose phosphate pathway and the TCA cycle.
320	PWY-1042 | ESCHERICHIA_COLI	Glycolysis IV (plant cytosol)	*Escherichia*	*Escherichia coli*	
321	PWY-1042 | FAECALIBAC-TERIUM_PRAUSNITZII	Glycolysis IV (plant cytosol)	*Faecalibac-terium*	*Faecalibac-terium prausnitzii*	

351	PWY-2942 | FAECALIBACTERIUM_PRAUSNITZII	L-lysine biosynthesis III	*Faecalibacterium*	*Faecalibacterium prausnitzii*	Six pathways are now recognized in bacteria, most algae, fungi and higher plants for the biosynthesis of lysine. They are divided into two groups - the diaminopimelate (DAP) pathways, and the L-2-aminoadipate pathways.
355	PWY-2942 | LACHNOSPIRACEAE_BACTERIUM_5_1_63FAA	L-lysine biosynthesis III	*Lachnospiraceae*	*Lachnospiraceae bacterium 5 1 63FAA*	
359	PWY-2942 | UNCLASSIFIED	L-lysine biosynthesis III	Unclassified	Unclassified	
362	PWY-3001 | UNCLASSIFIED	Superpathway of L-isoleucine biosynthesis I	Unclassified	Unclassified	L-isoleucine is almost always synthesized from 2-oxobutanoate. In the most common pathway, 2-oxobutanoate is generated from L-aspartate via L-threonine (this pathway). The key enzyme in this pathway is threonine deaminase, encoded by the ilvA gene.
370	PWY-3841 | PHOCAEICOLA_VULGATUS	Folate transformations II	*Phocaeicola*	*Phocaeicola vulgatus*	This pathway describes the formation of the formyl and methyl derivatives of tetrahydrofolate (vitamin B9).
391	PWY-5097 | ANAEROSTIPES_HADRUS	L-lysine biosynthesis VI	*Anaerostipes*	*Anaerostipes hadrus*	Six pathways are now recognized in bacteria, most algae, fungi and higher plants for the biosynthesis of lysine. They are divided into two groups - the diaminopimelate (DAP) pathways, and the L-2-aminoadipate pathways.
395	PWY-5097 | BACTEROIDES_STERCORIS	L-lysine biosynthesis VI	*Bacteroides*	*Bacteroides stercoris*	
413	PWY-5097 | UNCLASSIFIED	L-lysine biosynthesis VI	Unclassified	Unclassified	
419	PWY-5100 | UNCLASSIFIED	Pyruvate fermentation to acetate and lactate II	Unclassified	Unclassified	With glucose as a substrate, lactate, acetate, carbon dioxide and hydrogen are produced as the main fermentation products. Glucose degradation to pyruvate occurs via the conventional Embden-Meyerhof pathway.
428	PWY-5103 | UNCLASSIFIED	L-isoleucine biosynthesis III	Unclassified	Unclassified	L-isoleucine is almost always synthesized from 2-oxobutanoate. In the most common pathway, 2-oxobutanoate is generated from L-aspartate via L-threonine. The key enzyme in that pathway is threonine deaminase.
429	PWY-5104 | ANAEROSTIPES_HADRUS	L-isoleucine biosynthesis IV	*Anaerostipes*	*Anaerostipes hadrus*	In addition to the standard pathway, microorganisms have developed several alternative pathways for L-isoleucine biosynthesis.

442	PWY-5188 | FLAVONIFRAC-TOR_PLAUTII	Tetrapyrrole biosynthesis I (from glutamate)	*Flavonifractor*	*Flavonifractor plautii*	Tetrapyrroles function as a metal-binding cofactor in many important enzymes, proteins and pigments, such as heme, chlorophyll, cobalamine (vitamin B12), siroheme, and cofactor F430.
461	PWY-5659 | ANAEROSTIPES_HADRUS	GDP-mannose biosynthesis	*Anaerostipes*	*Anaerostipes hadrus*	GDP-$alpha$-D-mannose is a key substrate in glycoprotein formation
462	PWY-5659 | BACTEROIDES_OVATUS	GDP-mannose biosynthesis	*Bacteroides*	*Bacteroides ovatus*	
466	PWY-5659 | FAECALIBAC-TERIUM_PRAUSNITZII	GDP-mannose biosynthesis	*Faecalibac-terium*	*Faecalibac-terium prausnitzii*	
472	PWY-5667 | ANAEROSTIPES_HADRUS	CDP-diacylglycerol biosynthesis I	*Anaerostipes*	*Anaerostipes hadrus*	Phospholipids are important membrane components. Most of the phospholipids belong to the category of phospho-glycerides. The simplest phosphoglycerides, which are known as phosphatidates, are composed of a glycerol molecule attached to two fatty acids and one phosphate group.
477	PWY-5667 | BAC-TEROIDES_UNIFORMIS	CDP-diacylglycerol biosynthesis I	*Bacteroides*	*Bacteroides uniformis*	
499	PWY-5667 | UNCLASSIFIED	CDP-diacylglycerol biosynthesis I	Unclassified	Unclassified	
500	PWY-5676 | UNCLASSIFIED	Acetyl-CoA fermentation to butanoate II	Unclassified	Unclassified	In this pathway, acetyl-CoA, which is derived from ethanol (see ethanol degradation I) can be processed in two routes.
517	PWY-5686 | FAECALIBAC-TERIUM_PRAUSNITZII	UMP biosynthesis	*Faecalibac-terium*	*Faecalibac-terium prausnitzii*	Pyrimidine and purine nucleoside triphosphates are the activated precursors of DNA and RNA.
525	PWY-5686 | UNCLASSIFIED	UMP biosynthesis	Unclassified	Unclassified	
538	PWY-5695 | FAECALIBAC-TERIUM_PRAUSNITZII	Urate biosynthesis/inosine 5'-phosphate degradation	*Faecalibac-terium*	*Faecalibac-terium prausnitzii*	It is part of the degradation of purines, which is found in many organisms.
544	PWY-5695 | UNCLASSIFIED	Urate biosynthesis/inosine 5'-phosphate degradation	Unclassified	Unclassified	
565	PWY-6121 | ANAEROSTIPES_HADRUS	5-aminoimidazole ribonucleotide biosynthesis I	*Anaerostipes*	*Anaerostipes hadrus*	5-amino-1-(5-phospho-β-D-ribosyl)imidazole (AIR) is a key intermediate in the biosynthesis of purine nucleotides and thiamine.
571	PWY-6121 | ENTERO-CLOSTER_BOLTEAE	5-aminoimidazole ribonucleotide biosynthesis I	*Enterocloster*	*Enterocloster bolteae*	
579	PWY-6121 | FAECALIBAC-TERIUM_PRAUSNITZII	5-aminoimidazole ribonucleotide biosynthesis I	*Faecalibac-terium*	*Faecalibac-terium prausnitzii*	

580	PWY-6121 | FLAVONIFRACTOR_PLAUTII	5-aminoimidazole ribonucleotide biosynthesis I	*Flavonifractor*	*Flavonifractor plautii*	
583	PWY-6121 | ODORIBACTER_SPLACHNICUS	5-aminoimidazole ribonucleotide biosynthesis I	*Odoribacter*	*Odoribacter splachnicus*	
584	PWY-6121 | PARASUTTERELLA_EXCREMENTIHOMINIS	5-aminoimidazole ribonucleotide biosynthesis I	*Parasutterella*	*Parasutterella excrementihominis*	
569	PWY-6121 | RUMINOCOCCUS_TORQUES	5-aminoimidazole ribonucleotide biosynthesis I	*Blautia*	*Ruminococcus torques*	
599	PWY-6122 | ENTEROCLOSTER_BOLTEAE	5-aminoimidazole ribonucleotide biosynthesis II	*Enterocloster*	*Enterocloster bolteae*	5-amino-1-(5-phospho-β-D-ribosyl)imidazole (AIR) is a key intermediate in the biosynthesis of purine nucleotides and thiamine
610	PWY-6122 | FAECALIBACTERIUM_PRAUSNITZII	5-aminoimidazole ribonucleotide biosynthesis II	*Faecalibacterium*	*Faecalibacterium prausnitzii*	
611	PWY-6122 | FLAVONIFRACTOR_PLAUTII	5-aminoimidazole ribonucleotide biosynthesis II	*Flavonifractor*	*Flavonifractor plautii*	
615	PWY-6122 | PARASUTTERELLA_EXCREMENTIHOMINIS	5-aminoimidazole ribonucleotide biosynthesis II	*Parasutterella*	*Parasutterella excrementihominis*	
597	PWY-6122 | RUMINOCOCCUS_TORQUES	5-aminoimidazole ribonucleotide biosynthesis II	*Blautia*	*Ruminococcus torques*	
622	PWY-6123 | BACTEROIDES_FRAGILIS	Inosine-5'-phosphate biosynthesis I	*Bacteroides*	*Bacteroides fragilis*	De novo biosynthesis of purines starts with the synthesis of inosine-5'-phosphate (IMP), which can be converted to all other purines.
625	PWY-6123 | BACTEROIDES_XYLANISOLVENS	Inosine-5'-phosphate biosynthesis I	*Bacteroides*	*Bacteroides xylanisolvens*	
632	PWY-6124 | BACTEROIDES_OVATUS	Inosine-5'-phosphate biosynthesis II	*Bacteroides*	*Bacteroides ovatus*	De novo biosynthesis of purines starts with the synthesis of inosine-5'-phosphate (IMP), which can be converted to all other purines.
658	PWY-6151 | BACTEROIDES_UNIFORMIS	S-adenosyl-L-methionine cycle I	*Bacteroides*	*Bacteroides uniformis*	About 20% of the L-methionine pool is used as a building block of proteins. The rest is converted to S-adenosyl-L-methionine (SAM), the major methyl donor in the cell.
670	PWY-6151 | ROSEBURIA_INTESTINALIS	S-adenosyl-L-methionine cycle I	*Roseburia*	*Roseburia intestinalis*	
671	PWY-6151 | ROSEBURIA_INULINIVORANS	S-adenosyl-L-methionine cycle I	*Roseburia*	*Roseburia inulinivorans*	

660	PWY-6151 | BLAUTIA_OBEUM	S-adenosyl-L-methionine cycle I	*Blautia*	*Blautia obeum*	
672	PWY-6151 | UNCLASSIFIED	S-adenosyl-L-methionine cycle I	Unclassified	Unclassified	
680	PWY-6163 | DOREA_LONGICATENA	Chorismate biosynthesis from 3-dehydroquinate	Dorea	Dorea longicatena	In this pathway chorismate is synthesized from 3-dehydroquinate.
697	PWY-621 | ESCHERICHIA_COLI	Sucrose degradation III (sucrose invertase)	*Escherichia*	*Escherichia coli*	In this pathway sucrose is cleaved by invertase
713	PWY-6277 | ENTERO-CLOSTER_BOLTEAE	Superpathway of 5-aminoimidazole ribonucleotide biosynthesis	*Enterocloster*	*Enterocloster bolteae*	5-amino-1-(5-phospho-β-D-ribosyl)imidazole (AIR) is a key intermediate in the biosynthesis of purine nucleotides and thiamine.
724	PWY-6277 | FAECALIBAC-TERIUM_PRAUSNITZII	Superpathway of 5-aminoimidazole ribonucleotide biosynthesis	*Faecalibac-terium*	*Faecalibac-terium prausnitzii*	
725	PWY-6277 | FLAVONIFRAC-TOR_PLAUTII	Superpathway of 5-aminoimidazole ribonucleotide biosynthesis	*Flavonifractor*	*Flavonifractor plautii*	
729	PWY-6277 | PARASUTTERELLA_EX-CREMENTIHOMINIS	Superpathway of 5-aminoimidazole ribonucleotide biosynthesis	*Parasutterella*	*Parasutterella excrementiho-mini*	
711	PWY-6277 | RUMINOCOC-CUS_TORQUES	Superpathway of 5-aminoimidazole ribonucleotide biosynthesis	*Blautia*	*Ruminococcus torques*	
740	PWY-6305 | FAECALIBAC-TERIUM_PRAUSNITZII	Putrescine biosynthesis IV	*Faecalibac-terium*	*Faecalibac-terium prausnitzii*	This pathway produces the polyamine putrescine. This metabolite like other polyamines is cationic and may be involved in interactions with proteins, DNA and RNA molecules.
748	PWY-6317 | FAECALIBAC-TERIUM_PRAUSNITZII	Galactose degradation I (Leloir pathway)	*Faecalibac-terium*	*Faecalibac-terium prausnitzii*	The enzymes of the Leloir pathway (here named D-galactose degradation I (Leloir pathway)) catalyze the conversion of D-galactose to the more metabolically versatile D-glucopyranose 6-phosphate. This pathway is required since galactose itself cannot be used for glycolysis directly.
751	PWY-6317 | UNCLASSIFIED	Galactose degradation I (Leloir pathway)	Unclassified	Unclassified	
753	PWY-6353 | UNCLASSIFIED	Purine nucleotides degradation II (aerobic)	Unclassified	Unclassified	This pathway depicts the degradation of purine nucleotides to purine nucleosides, purine bases, and urate.
756	PWY-6385 | BAC-TEROIDES_XYLANISOLVENS	Peptidoglycan biosynthesis III (mycobacteria)	*Bacteroides*	*Bacteroides xylanisolvens*	Peptidoglycan is best described as a fisherman's net, with each cell of the mesh defined by two segments of parallel, glycan threads, held together by two small elastic peptide crosslinks, which allow the net to expand or shrink.

794	PWY-6387 | BACTEROIDES_UNIFORMIS	UDP-N-acetylmuramoyl-pentapeptide biosynthesis I (meso-diaminopimelate containing)	*Bacteroides*	*Bacteroides uniformis*	Peptidoglycan is best described as a fisherman's net, with each cell of the mesh defined by two segments of parallel, glycan threads, held together by two small elastic peptide crosslinks, which allow the net to expand or shrink.
819	PWY-6527 | FAECALIBACTERIUM_PRAUSNITZII	Stachyose degradation	*Faecalibacterium*	*Faecalibacterium prausnitzii*	This pathway describes the degradation of stachyose and raffinose into α-D-galactopyranose.
821	PWY-6527 | UNCLASSIFIED	Stachyose degradation	Unclassified	Unclassified	.
838	PWY-6609 | ESCHERICHIA_COLI	Adenine and adenosine salvage III	*Escherichia*	*Escherichia coli*	Adenosine nucleotides can be synthesized de novo. In that route adenosine-monophosphate (AMP) is synthesized via inosinate (IMP) and adenylosuccinate, which is converted to AMP by the action of adenylosuccinate lyase.
836	PWY-6609 | RUMINOCOCCUS_GNAVUS	Adenine and adenosine salvage III	*Ruminococcus*	*Ruminococcus gnavus*	
842	PWY-6609 | UNCLASSIFIED	Adenine and adenosine salvage III	Unclassified	Unclassified	
1222	PWY66-422 | FAECALIBACTERIUM_PRAUSNITZII	D-galactose degradation V (Leloir pathway)	*Faecalibacterium*	*Faecalibacterium prausnitzii*	The enzymes of the Leloir pathway catalyze the conversion of D-galactose to the more metabolically versatile D-glucopyranose 6-phosphate. This pathway is required since galactose itself cannot be used for glycolysis directly.
1225	PWY66-422 | UNCLASSIFIED	D-galactose degradation V (Leloir pathway)	Unclassified	Unclassified	
847	PWY-6700 | ANAEROSTIPES_HADRUS	Queuosine biosynthesis	*Anaerostipes*	*Anaerostipes hadrus*	Queuosine is an important 7-deazapurine modified nucleoside that is present in certain tRNAs in bacteria and most eukaryotes (with the exception of mycoplasmas and yeast).
855	PWY-6700 | PHOCAEICOLA_VULGATUS	Queuosine biosynthesis	*Phocaeicola*	*Phocaeicola vulgatus*	
865	PWY-6700 | UNCLASSIFIED	Queuosine biosynthesis	Unclassified	Unclassified	
873	PWY-6703 | BACTEROIDES_UNIFORMIS	PreQ0 biosynthesis	*Bacteroides*	*Bacteroides uniformis*	7-Deazapurines are compounds that contain pyrrolopyrimidine functional groups (similar to purines, but lacking one of the nitrogens in the pentameric ring). These compounds form a structurally diverse class of nucleoside analogues that often possess antibiotic, antineoplastic, or antiviral activities.
875	PWY-6703 | BACTEROIDES_XYLANISOLVENS	PreQ0 biosynthesis	*Bacteroides*	*Bacteroides xylanisolvens*	

877	PWY-6703 | ESCHERICHIA_COLI	PreQ0 biosynthesis	*Escherichia*	*Escherichia coli*	
878	PWY-6703 | LACHNOSPIRA-CEAE_BACTERIUM_5_1_63FAA	PreQ0 biosynthesis	*Lachnospiraceae*	*Lachnospiraceae bacterium 5 1 63FAA*	
891	PWY-6737 | CLOSTRIDIUM_LEPTUM	Starch degradation V	*Clostridium*	*Clostridium leptum*	Many organisms including bacteria, fungi, metazoa, and plants can degrade glucose polymers derived from starch or glycogen.
895	PWY-6737 | DOREA_LONGICATENA	Starch degradation V	*Dorea*	*Dorea longicatena*	
896	PWY-6737 | ESCHERICHIA_COLI	Starch degradation V	*Escherichia*	*Escherichia coli*	
901	PWY-6737 | FAECALIBAC-TERIUM_PRAUSNITZII	Starch degradation V	*Faecalibacterium*	*Faecalibacterium prausnitzii*	
913	PWY-6897 | UNCLASSIFIED	Thiamin salvage II	Unclassified	Unclassified	Thiamine diphosphate, also known as vitamin B1, is known to play a fundamental role in energy metabolism. It is an essential cofactor for a variety of enzymes.
950	PWY-7111 | RUMINOCOC-CUS_BROMII	Pyruvate fermentation to isobutanol (engineered)	*Ruminococcus*	*Ruminococcus bromii*	Higher alcohols, such as isobutanol, possess several beneficial characteristics, such as a low hygroscopicity, vapor pressure and corrosivity.
963	PWY-7199 | ESCHERICHIA_COLI	Pyrimidine deoxyribonucleosides salvage	*Escherichia*	*Escherichia coli*	Deoxyribonucleotides are synthesised de novo at the diphosphate level through reduction of the 2'-hydroxyl group of the corresponding ribonucleotides.
969	PWY-7208 | EUBAC-TERIUM_RECTALE	Superpathway of pyrimidine nucleobases salvage	*Eubacterium*	*Eubacterium rectale*	The essential ribonucleoside triphosphates (UTP and CTP) can be synthesized either de novo or by utilizing free pyrimidine bases (uracil and cytosine) or their nucleosides (uridine and cytidine) by importing them from the environment.
971	PWY-7208 | PARASUTTERELLA_EX-CREMENTIHOMINIS	Superpathway of pyrimidine nucleobases salvage	*Parasutterella*	*Parasutterella excrementihominis*	
988	PWY-7219 | BAC-TEROIDES_UNIFORMIS	Adenosine ribonucleotides de novo biosynthesis	*Bacteroides*	*Bacteroides uniformis*	The first purine nucleotide that is synthesized de novo is inosine monophosphate (IMP). IMP is converted to adenylosuccinate by the enzyme adenylosuccinate synthase, and the latter is converted to the first adenosine nucleotide, adenosine 5'-monophosphate (AMP) by the action of adenylosuccinate lyase.

1003	PWY-7219 | DOREA_FORMICIGE-NERANS	Adenosine ribonucleotides de novo biosynthesis	*Dorea*	*Dorea formicigenerans*	
1007	PWY-7219 | ANAEROBU-TYRICUM_HALLII	Adenosine ribonucleotides de novo biosynthesis	*Anaerobutyricum*	*Anaerobutyricum hallii*	
1008	PWY-7219 | EUBAC-TERIUM_RECTALE	Adenosine ribonucleotides de novo biosynthesis	*Eubacterium*	*Eubacterium rectale*	
1027	PWY-7220 | PHOCAE-ICOLA_VULGATUS	Adenosine deoxyribonucleotides de novo biosynthesis II	*Phocaeicola*	*Phocaeicola vulgatus*	In this pathway, adenosine pyrophosphate is converted in its deoxy form.
1044	PWY-7221 | BIFIDOBAC-TERIUM_LONGUM	Guanosine ribonucleotides de novo biosynthesis	*Bifidobacterium*	*Bifidobacterium longum*	The first purine nucleotide that is synthesized de novo is inosine-5'-phosphate (IMP). IMP is converted to xanthosine monophosphate XMP by the enzyme inosine 5'-monophosphate dehydrogenase, and the latter is converted to the first guanosine nucleotide, GMP, by the action of GMP synthetase, an enzyme that can use either glutamine or ammonia as substrate.
1042	PWY-7221 | PHOCAE-ICOLA_VULGATUS	Guanosine ribonucleotides de novo biosynthesis	*Phocaeicola*	*Phocaeicola vulgatus*	
1047	PWY-7221 | RUMINOCOC-CUS_TORQUES	Guanosine ribonucleotides de novo biosynthesis	*Blautia*	*Ruminococcus torques*	
1068	PWY-7222 | PHOCAE-ICOLA_VULGATUS	Guanosine deoxyribonucleotides de novo biosynthesis II	*Phocaeicola*	*Phocaeicola vulgatus*	Once the ribonucleoside GDP is formed, it can be reduced to the deoxy form dGDP by the enzyme ribonucleoside diphosphate reductase.
1074	PWY-7228 | PHOCAE-ICOLA_VULGATUS	Superpathway of guanosine nucleotides de novo biosynthesis I	*Phocaeicola*	*Phocaeicola vulgatus*	The first purine nucleotide that is synthesized de novo is inosine-5'-phosphate (IMP). IMP is converted to xanthosine monophosphate (XMP) by the enzyme inosine 5'-monophosphate dehydrogenase, and the latter is converted to the first guanosine nucleotide, GMP, by the action of GMP synthetase, an enzyme that can use either glutamine or ammonia as the nitrogen donor.
1093	PWY-724 | UNCLASSIFIED	Superpathway of L-lysine, L-threonine and L-methionine biosynthesis II	Unclassified	Unclassified	In this superpathway, L-lysine, L-threonine and L-methionine are synthesized from L-aspartate.
1090	PWY-7242 | FAECALIBAC-TERIUM_PRAUSNITZII	D-fructuronate degradation	*Faecalibacterium*	*Faecalibacterium prausnitzii*	Multiple degradation pathways lead to the metabolite D-fructuronate.
1096	PWY-7282 | BAC-TEROIDES_FRAGILIS	4-amino-2-methyl-5-phosphomethylpyrimidine biosynthesis (yeast)	*Bacteroides*	*Bacteroides fragilis*	The formation of the two heterocycles involved in the biosynthesis of thiamine diphosphate (vitamin B1), i.e. the thiazole and pyrimidine moiety.

1097	PWY-7282 | BACTEROIDES_OVATUS	4-amino-2-methyl-5-phosphomethylpyrimidine biosynthesis (yeast)	*Bacteroides*	*Bacteroides ovatus*	
1108	PWY-7357 | ROSEBURIA_INTESTINALIS	Thiamin formation from pyrithiamine and oxythiamine (yeast)	*Roseburia*	*Roseburia intestinalis*	Thiamine (vitamin B1) and its biological active derivate thiamine diphosphate is synthesized in prokaryotes, yeasts and plants utilizing complex pathways.
1112	PWY-7383 | UNCLASSIFIED	Anaerobic energy metabolism (invertebrates, cytosol)	Unclassified	Unclassified	Some facultative anaerobes may contain either mitochondria that function anaerobically, or hydrogenosomes as cellular organelles in which reactions of anaerobic respiration occur.
1250	RHAMCAT-PWY | ROSEBURIA_INTESTINALIS	L-rhamnose degradation I	*Roseburia*	*Roseburia intestinalis*	L-rhamnose is a deoxy-hexose sugar commonly found in plants as a part of complex pectin polysaccharides and in many bacteria as a common component of the cell wall.
1253	SALVADEHYPOX-PWY | RUMINOCOCCUS_TORQUES	Adenosine nucleotides degradation II	*Blautia*	*Ruminococcus torques*	Degradation pathways start with the nucleotide forms and convert them to simpler forms.
1261	SER-GLYSYN-PWY | FAECALIBACTERIUM_PRAUSNITZII	Superpathway of L-serine and glycine biosynthesis I	*Faecalibacterium*	*Faecalibacterium prausnitzii*	Serine biosynthesis is a major metabolic pathway in many organisms. L-serine is not only used in protein synthesis, but also as a precursor for the biosynthesis of glycine, cysteine, tryptophan, and phospholipids.
1265	SER-GLYSYN-PWY | RUMINOCOCCUS_BROMII	Superpathway of L-serine and glycine biosynthesis I	*Ruminococcus*	*Ruminococcus bromii*	
1284	THRESYN-PWY | UNCLASSIFIED	Superpathway of L-threonine biosynthesis	Unclassified	Unclassified	The overall superpathway of threonine biosynthesis as shown here covers the entire process of converting the central energy metabolism molecule oxaloacetate into L-threonine.
1285	TRNA-CHARGING-PWY | UNCLASSIFIED	tRNA charging	Unclassified	Unclassified	This pathway groups together all *Escherichia coli* tRNA charging reactions.
1298	VALSYN-PWY | ANAEROSTIPES_HADRUS	L-valine biosynthesis	*Anaerostipes*	*Anaerostipes hadrus*	The pathway of valine biosynthesis is a four-step pathway that shares all of its steps with the parallel pathway of isoleucine biosynthesis. These entwined pathways are part of the superpathway of branched chain amino acid biosynthesis, that generates not only isoleucine and valine, but also leucine.
1309	VALSYN-PWY | ENTEROCLOSTER_BOLTEAE	L-valine biosynthesis	*Enterocloster*	*Enterocloster bolteae*	
1312	VALSYN-PWY | DOREA_FORMICIGENERANS	L-valine biosynthesis	*Dorea*	*Dorea formicigenerans*	

Table S1. Nodes map of the nodes being mentioned in the manuscript. The descriptions of the pathways are extracted from ByoCyc[1] and MetaCyc[2].

References

1. Karp, P. D. *et al.* The biocyc collection of microbial genomes and metabolic pathways. *Briefings bioinformatics* **20**, 1085–1093 (2019).

2. Caspi, R. *et al.* The MetaCyc database of metabolic pathways and enzymes and the BioCyc collection of Pathway/Genome Databases. *Nucleic Acids Res.* **42**, D459–D471, DOI: 10.1093/nar/gkt1103 (2013). https://academic.oup.com/nar/article-pdf/42/D1/D459/3578456/gkt1103.pdf.
